# Development and application of the human intestinal tract chip, a phylogenetic microarray: analysis of universally conserved phylotypes in the abundant microbiota of young and elderly adults

**DOI:** 10.1111/j.1462-2920.2009.01900.x

**Published:** 2009-07

**Authors:** Mirjana Rajilić-Stojanović, Hans G H J Heilig, Douwe Molenaar, Kajsa Kajander, Anu Surakka, Hauke Smidt, Willem M de Vos

**Affiliations:** 1 Laboratory of Microbiology, Wageningen UniversityDreijenplein 10, 6703 HB Wageningen, the Netherlands; 2 NIZO Food ResearchP.O Box 20, 6710 BA Ede, the Netherlands; 3 Valio Ltd, R and DMeijeritie 4, 00370 Helsinki, Finland; 4 Department of Basic Veterinary Sciences, University of HelsinkiPO Box 66, 00014, Finland

## Abstract

In this paper we present the *in silico* assessment of the diversity of variable regions of the small subunit ribosomal RNA (SSU rRNA) gene based on an ecosystem-specific curated database, describe a probe design procedure based on two hypervariable regions with minimal redundancy and test the potential of such probe design strategy for the design of a flexible microarray platform. This resulted in the development and application of a phylogenetic microarray for studying the human gastrointestinal microbiota – referred as the human intestinal tract chip (HITChip). Over 4800 dedicated tiling oligonucleotide probes were designed based on two hypervariable regions of the SSU rRNA gene of 1140 unique microbial phylotypes (< 98% identity) following analysis of over 16 000 human intestinal SSU rRNA sequences. These HITChip probes were hybridized to a diverse set of human intestinal samples and SSU rRNA clones to validate its fingerprinting and quantification potential. Excellent reproducibility (median Pearson's correlation of 0.99) was obtained following hybridization with T7 polymerase transcripts generated *in vitro* from SSU rRNA gene amplicons. A linear dose–response was observed with artificial mixtures of 40 different representative amplicons with relative abundances as low as 0.1% of total microbiota. Analysis of three consecutively collected faecal samples from ten individuals (five young and five elderly adults) revealed temporal dynamics and confirmed that the adult intestinal microbiota is an individual-specific and relatively stable ecosystem. Further analysis of the stable part allowed for the identification of a universal microbiota core at the approximate genus level (90% sequence similarity). This core consists of members of *Actinobacteria*, *Bacteroidetes* and *Firmicutes*. Used as a phylogenetic fingerprinting tool with the possibility for relative quantification, the HITChip has the potential to bridge the gaps in our knowledge in the quantitative and qualitative description of the human gastrointestinal microbiota composition.

## Introduction

The human gastrointestinal tract is densely populated by a complex microbial ecosystem, which consists of thousands of microbial species-level phylogenetic types (phylotypes) (Rajilić-Stojanović*et al*., 2007). The human microbiota primarily contributes to the digestion but is also important for health and well-being (Zoetendal *et al*., 2006a). Because of its relevance, the human intestinal microbiota composition and activity have been extensively studied in recent years (Gill *et al*., 2006; Palmer *et al*., 2007; Li *et al*., 2008; Scanlan *et al*., 2008). The expansion of these studies paralleled the development of various high throughput–omics technologies (Nicholson *et al*., 2005; Kurokawa *et al*., 2007).

Phylogenetic microarrays are among the leading comprehensive molecular techniques that enable high-throughput analysis of microbial ecosystems and can be used for strain typing, the determination of diversity and the analysis of the functionality of microbial ecosystems (Guschin *et al*., 1997; Gentry *et al*., 2006; DeSantis *et al*., 2007; Wagner *et al*., 2007). These phylogenetic microarrays are in most cases based on the small subunit ribosomal RNA (SSU rRNA) gene (Bodrossy and Sessitsch, 2004). Such microarrays can be useful for the characterization of human intestinal microbiota composition and dynamics as more than 60% of the currently known diversity of gut microbiota phylotypes has only been detected by 16S rRNA gene sequences, whereas less than 40% was found by cultivation (Rajilić-Stojanović*et al*., 2007). This situation resembles the situation reported for many other complex microbial ecosystems (Schloss and Handelsman, 2004). A recent application of a comprehensive SSU rRNA gene-based phylogenetic microarray showed that this technology provides superior diagnostic power for the analysis of the microbial community structure when compared with the clone library approach (DeSantis *et al*., 2007).

In this paper we present an oligonucleotide probe design strategy based on two hypervariable regions of the SSU rRNA gene. This approach is high throughput, can produce probes with similar predicted hybridization behaviour and allows easy addition of probes that target newly reported inhabitants of the ecosystem of interest. The potential of this probe design strategy for phylogenetic microarrays was tested for the development and application of the HITChip (human intestinal tract chip). The results show that the HITChip is a highly reproducible phylogenetic fingerprinting tool that can be used for relative quantification of microbial groups. Analysis of the adult microbiota in time allowed for identification of a universal microbiota core, consisting of members of *Actinobacteria*, *Bacteroidetes* and *Firmicutes*, which however, shows no common phylotypes.

## Results and discussion

### Development of the HITChip – identification of hypervariable SSU rRNA regions and probe design

A phylogenetic microarray was developed for determining the microbial diversity in the human intestinal tract. The selected approach consisted of assessing the diversity of variable regions of the SSU rRNA gene based on a curated database, developing a probe design procedure based on two hypervariable regions with minimal redundancy, and addressing the potential of such probe design strategy for the design of a flexible microarray platform.

Phylogenetic microarrays, similar to any SSU rRNA gene-based technique, primarily depend on the design of probes at different levels of specificity. An accurate design of universal or phylotype-specific signature sequences within the SSU rRNA gene would require a complete database of all SSU rRNA sequences. However, as the microbial world is still largely unknown, this approach is not yet feasible. Therefore, it was decided to design an ecosystem-specific phylogenetic microarrays. This strategy can potentially be applied to any ecosystem, but the human intestinal microbiota was selected as it has considerable complexity, shows significant coverage of SSU rRNA sequences in public databases and is subject to intensive studies (Eckburg *et al*., 2005; Ley *et al*., 2006; Leitch *et al*., 2007; Li *et al*., 2008). The resulting phylogenetic microarray was designated the HITChip and is described below.

Following the redesign of universal primer sets to optimize universal amplification of bacterial sequences (Watanabe *et al*., 2001), it was found that most of these matched maximally 74% of the presently available SSU rRNA gene sequences (Horz *et al*., 2005). Furthermore, the specific probe design for comprehensive coverage of the occurring microbial diversity is a laborious process and therefore feasible only for ecosystems of limited complexity. One alternative to the conventional probe design is the tiling of the entire gene sequence (Chee *et al*., 1996), selection of a subset of the most distinct tiling probes (Palmer *et al*., 2006; DeSantis *et al*., 2007; Palmer *et al*., 2007) or probe design focused on a specific hypervariable region of the SSU rRNA gene (Wilson *et al*., 2002). Neither of these high-throughput probe design strategies has the capacity to target all of the more than 700 000 SSU rRNA gene sequences present in today's databases (http://www.arb-silva.de), which shows the need for a design of ecosystem-specific phylogenetic microarrays.

To design the HITChip, the sequences of all nine SSU rRNA variable regions were extracted from a database (last update December 2006) of the sequences retrieved from the human intestinal tract that was embedded in the ARB working environment (Ludwig *et al*., 2004). The database contained approximately 16 000 SSU rRNA gene sequences that were grouped into 1140 distinct phylotypes based on a threshold of 98% sequence identity (Table S1) (Rajilić-Stojanović*et al*., 2007). Approximately 400 phylotypes were represented by full-length sequences, while the remaining ∼740 phylotypes were associated with partial sequences. A variable number of sequences per variable region, ranging from 446 to 1107, could be extracted from this database (Table 1). Close inspection of these sequences showed that the variable regions V1 and V2 at the start of the SSU gene and V6, V8 and V9 at the end of the SSU gene contained the highest fraction (more than 80%) of unique sequences and could be considered as hypervariable. From these, the V1 and V6 regions showed the highest hypervariability and hence were selected for further analysis. Merging the sequence information of the V1 and V6 regions dramatically reduced the proportion of redundant sequences from 12.5% and 15.5% for the individual V1 and V6 regions, respectively, to 3.4% for the combined data. Only 63 phylotypes (5% of the total data set) were found to share identical V1 or V6 regions with other phylotypes, whereas there were only 12 pairs of phylotypes that shared identical sequences of both the V1 and the V6 regions. The latter 12 pairs were found to be highly similar along the entire SSU rRNA gene sequence, with an average pair-wise similarity of 99.4%, suggesting that they represent the same phylotype (Table S2). This analysis indicated that the merged sequence information of the V1 and V6 regions contained sufficient sequence information to discriminate between intestinal phylotypes. These results expand previous findings on the hypervariability of the V1 and the V6 regions with a limited set of SSU rRNA sequences (Heuer *et al*., 1999; Bertilsson *et al*., 2002).

**Table 1:** Position and diversity of variable regions of the SSU rRNA gene extracted based on the SSU rRNA gene sequence of 1140 human gastrointestinal phylotypes.

Variable region	Starting and ending positions (*E. coli*)	Reference	Number of sequences	Number of distinct sequences	Percentage of distinct sequences
V1	65–104	Bertilsson *et al*. (2002)	849	743	87.51
V2	174–235	Lane (1991)	875	759	86.74
V3	442–492	Lane (1991); Hugenholtz *et al*. (1998)	1107	687	62.06
V4	705–763	Hugenholtz *et al*. (1998)	1002	556	55.49
V5	822–879	Lane (1991); Hugenholtz *et al*. (1998)	988	682	69.03
V6	985–1047	Heuer *et al*. (1999)	895	754	84.25
V7	1115–1175	Roest (2007)	874	635	72.65
V8	1253–1313	Nübel *et al*. (1996)	865	699	80.81
V9	1420–1481	Lane (1991)	446	365	81.84

The feasibility to use the V1 and the V6 variable regions for phylotype-specific identification was initially confirmed by specific hybridizations in a dot-blot macroarray set up (data not shown). Because of considerable variation in length, ranging from 24 to 82 nucleotides, and with the goal to allow for uniform hybridization behaviour, subsequently a series of 24 nt tiling oligonucleotides was designed to cover the complete sequences of the V1 and V6 hypervariable regions of all 1140 phylotypes. This resulted in set of in total 4809 tiling oligonucleotides. Predicted melting temperatures (Tm) of these probes followed a unimodal symmetrical distribution over a wide range of temperatures, indicating that application of the probes in a single hybridization experiment was not feasible. Therefore, the oligonucleotide length was adjusted by addition or removal of a maximum of six nucleotides to either reduce or increase the predicted Tm. Using this procedure, 97% of all probes fitted a narrow Tm range of 70 ± 2°C. These improved probes were used for the production of a custom-synthesized Agilent array, the HITChip, to target the selected 1140 unique intestinal phylotypes (Rajilić-Stojanović*et al*., 2007). The number of intestinal phylotypes is steadily increasing, and the currently known number of uncultured phylotypes has been estimated to amount to around 1800 (Zoetendal *et al*., 2008). Based on the present database entries it can be calculated that the vast majority of the known phylotypes are covered on the HITChip platform described here at least at levels 1 and 2 (data not shown).

Using the probe design strategy described above, it can be expected that not all probes have distinct sequences. In fact, it could be calculated that from the 4809 HITChip probes, 3699 were unique (76.9%). Recently, another microarray platform for the rapid profiling of the gastrointestinal microbiota was reported (Palmer *et al*., 2006). The probe design strategy reported in that study was based on 359 gastrointestinal phylotypes found in intestinal samples of three individuals, and consisted of tiling the complete SSU rRNA gene and selecting the five most specific 40-nt-long probes for each targeted phylotype. Remarkably, the percentage of unique sequences in this microarray system (68.50%) was similar to the one obtained using the probe design strategy described here. Although the two approaches are not directly comparable as the length of the probes and the phylotype cut-off values were different, this result indicates that probe design based on two hypervariable segments of the SSU rRNA sequence will produce a similar proportion of distinct sequences when compared with the probe design strategy that takes into account the complete sequence of the target gene.

Subsequent to the probe design, the specificity of each HITChip probe was determined by *in silico* hybridization of the probe set against the total human intestinal microbiota SSU rRNA gene sequence database (Rajilić-Stojanović*et al*., 2007). For each probe, the lowest phylogenetic level that included all predicted hybridizing targets, was recorded as the specific phylogenetic level of the probe. Probes were categorized at three levels of increasing specificity: level 1, defined as order-like SSU rRNA gene sequence groups, level 2, defined as genus-like SSU rRNA gene sequence groups (sequence similarity > 90%), and level 3, phylotype-like SSU rRNA gene sequence groups (sequence similarity > 98%). Therefore, although all probes were designed to target unique phylotypes, some probe sequences were *a posteriori* assigned to different nodes in the phylogenetic tree (assigned to level 3, 60% of all probes; level 2, 29%; level 1, 9%). This strategy resulted in a similar outcome as that of the common hierarchical probe design strategies, with the distinction that probes specific for higher phylogenetic groups, in contrast to node probes, do not necessarily target all members of a phylogenetic group (Wagner *et al*., 2007). Similar to the hierarchical probe design strategies, an assigned probe specificity is not an absolute entity as it is influenced by the size and diversity of the SSU rRNA sequence space that is available at the time of probe design (Emrich *et al*., 2003; Li *et al*., 2005). The advantage of the approach presented here and other tiling approaches, however, is that probe specificity is assigned subsequent to the probe design, allowing for regular *in silico* re-assignment of probe specificity without the need for probe re-design.

### Validation of the HITChip – reproducibility assessment and use for identifying and quantifying intestinal phylotypes

Following the design and production of the HITChip containing 4809 tiling probes targeting 1140 intestinal phylotypes, the influence of the following three factors on the reproducibility of the readouts was tested: (i) DNA extraction, (ii) PCR amplification and *in vitro* transcription and (iii) microarray hybridization. The maximal reproducibility values of hybridization signals, determined as Pearson's coefficients, were 96.7% for different DNA extractions, 99.6% for different PCR amplifications and *in vitro* transcription, and 99.9% for different hybridizations (data not shown). In general, the reproducibility of data obtained with different PCR products was only slightly lower than those obtained with the same PCR product. Therefore, the standard procedure for sample hybridization included a single PCR with two independent hybridizations. The success of this approach can be illustrated by the observation that a median reproducibility of 98.9% has been obtained with over 800 samples that have been analysed so far on the HITChip (data not shown). The experimental variation in DNA extraction only slightly affected the hybridization results and the reproducibility of experiments performed with different DNA extracts was shown to be in the range of the technical reproducibility of expression microarrays (Larkin *et al*., 2005; Weis and Consortium, 2005).

It is well known that there is a strong variation of the hybridization signal intensity from probe–target duplexes with similar predicted hybridization behaviour (Loy *et al*., 2002; Bodrossy *et al*., 2003; Pozhitkov *et al*., 2006). Moreover, experimental studies with tiling oligonucleotide probes based on the SSU rRNA gene showed that the hybridization efficiency can vary more than 100-fold between probes (Palmer *et al*., 2006). This was confirmed in this study by hybridization of DNA of 40 known representative phylotypes that were derived from human intestinal clone libraries (Zoetendal *et al*., 1998; Suau *et al*., 1999) (data not shown). Due to this significant variation of the hybridization signal, the presence of a phylotype was defined based on the requirement that at least two of three probes per variable region should show a signal above the background. For the same reason, absolute quantification of individual phylotypes in a mixture is not feasible. However, the relative change of the hybridization signal of phylotype-specific probes was shown to be directly proportional to the quantitative change of the respective phylotype in a complex mixture consisting of the 40 previously selected phylotypes (Fig. S1). The experiments were performed with 10 phylotypes with relative concentrations of 0.1%, 0.3%, 1% and 3% in the mixture that contained 30 additional phylotypes at a constant concentration. These results showed that hybridization signals could be used as an indicator of changes in the relative abundance of a phylotype with the detection limit of 0.1% of the total SSU rRNA pool hybridized to the array. This is in line with previously reported detection thresholds (Palmer *et al*., 2006).

To further evaluate the application of the HITChip for quantification purposes, the influence of the number of PCR cycles on the readout was tested as this has been an issue for clone libraries or other phylogenetic arrays (Bonnet *et al*., 2002; Eckburg *et al*., 2005; Palmer *et al*., 2007). The results of the HITChip analysis of five samples generated using 20 and 35 PCR cycles appeared to be highly similar with an average similarity of 97.7% (SD 0.6%) (Fig. S2). This is in line with a recent study that showed absence of PCR bias when comparing two libraries constructed from PCR products amplified using 35 and 18 cycles (Acinas *et al*., 2005).

### Validation of the HITChip by comparison with FISH analysis of faecal samples

**Fig. 1:**
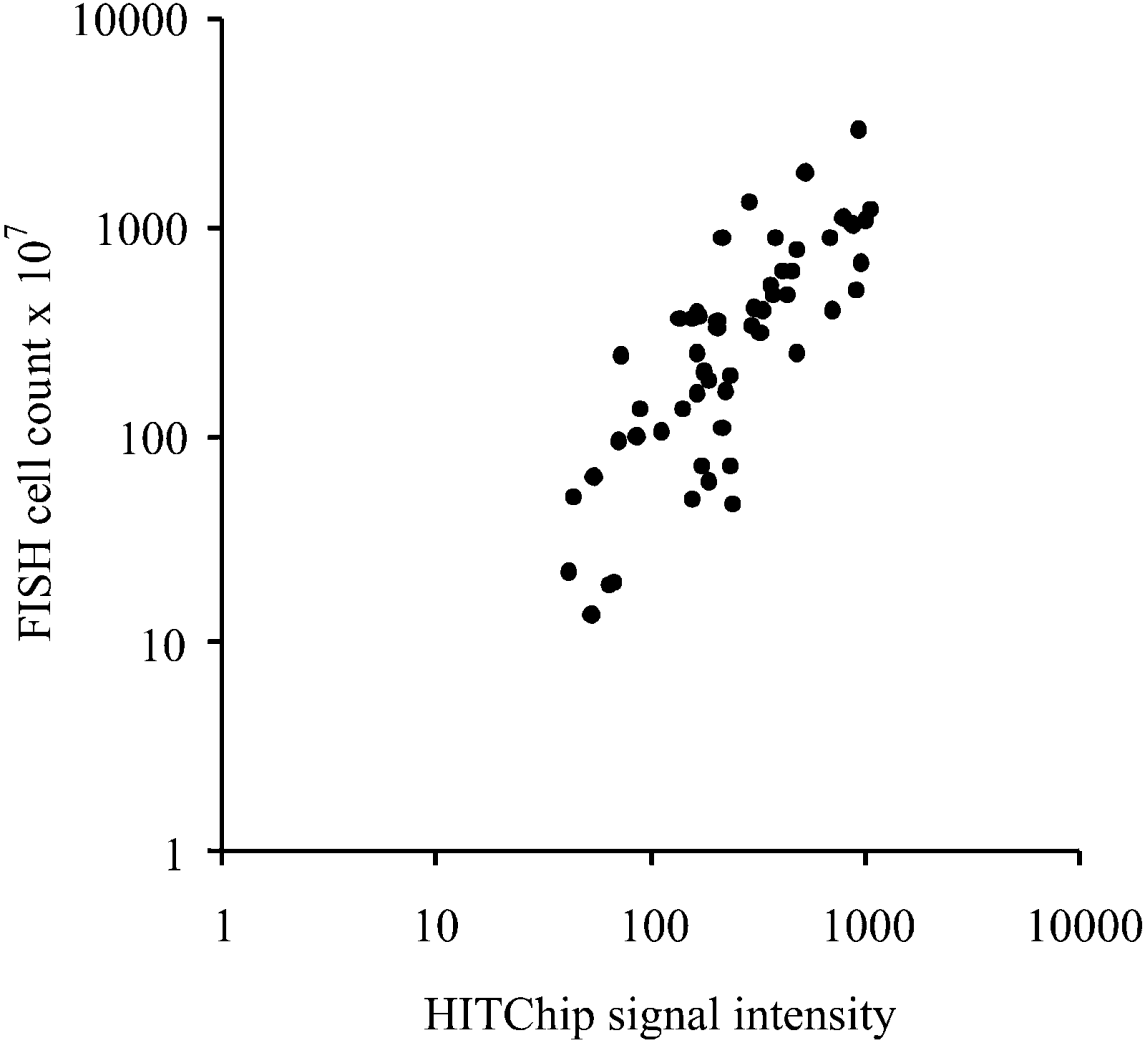
Correlation of results obtained by the *Bifidobacterium*-specific FISH quantification and sum of the *Bifidobacterium*-specific HITChip hybridization signals for 60 faecal samples. Faecal samples obtained from 20 individuals in three time points were analysed, of which four samples that did not reach detection limit with the FISH are not plotted on the graph.

**Fig. 2:**
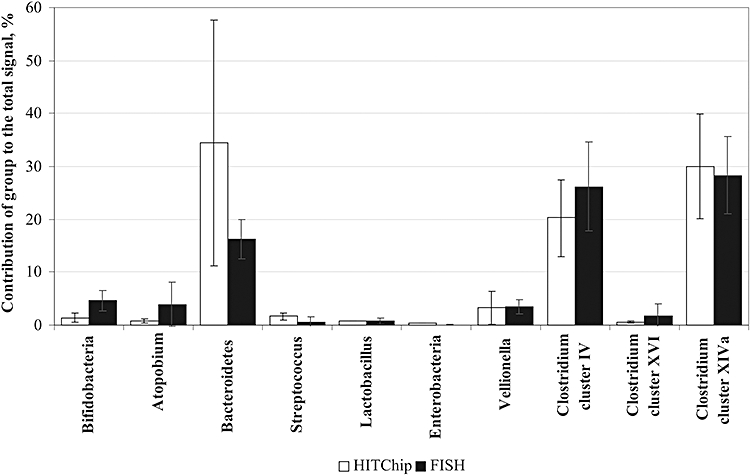
Correlation between relative abundance of ten phylogenetic groups assessed as average of the group-specific HITChip hybridization signals (empty columns) and FISH quantification (filled columns) based on the analysis of faecal microbiota of five subjects. Error bars indicate standard deviations.

Because of the considerable variation of the hybridization signal intensity among probe–target duplexes with similar predicted hybridization behaviour observed in this and other studies (Loy *et al*., 2002; Bodrossy *et al*., 2003; Pozhitkov *et al*., 2006), direct quantification of phylotypes was not feasible. Therefore, we tested the potential for quantification of genus-like (level 2) or higher phylogenetic groups. As a benchmark test case, we compared HITChip and FISH quantification of bifidobacteria in 60 faecal samples, which were collected periodically at three consecutive time intervals, from 20 elderly subjects (Fig. 1). A positive linear correlation was obtained between the two methods that showed a Pearson's correlation index of 0.72. An even improved correlation was obtained when comparing the relative increase or decrease of this group in time per individual [Pearson's correlation index of 0.93 (data not shown)], indicating that the HITChip platform has good quantification potential. Similar results were observed when quantitative data for members of the *Lactobacillus* genus obtained by quantitative PCR were compared with analyses using the HITChip (C. Booijink, unpubl. data). Furthermore, the averages of the HITChip hybridization signals calculated for 10 phylogenetic groups were compared with quantitative data obtained using FISH for faecal samples of five adult subjects (Lay *et al*., 2005). The relative quantitative data assessed with the HITChip correlated to a significant extent with the results of FISH (Fig. 2). The results for members of two dominant human intestinal groups, *Clostridium* cluster XIVa and *Clostridium* cluster IV, showed an almost complete correlation. Similarly, the HITChip data of other quantitatively less abundant groups, including *Veillonella* and *Lactobacillus,* correlated also very well with the FISH results. In contrast, the proportion of *Bacteroidetes* and streptococci as assessed by the HITChip was significantly higher than observed with FISH, while the opposite trend was observed for *Actinobacteria* that include the *Bifidobacterium* and *Atopobium* genera. As the HITChip analysis is performed based on DNA whereas the FISH analysis relies on quantification of cells by flow cytometry, differences in the number of SSU rRNA gene copies per cell can become a significant bias for DNA-based quantification of microbial groups. Various studies have shown that different bacterial groups are not equally active along the gastrointestinal tract (see Ben-Amor *et al*., 2005). This may result in more pronounced differences as the number of genome copies per cell is related to the growth rate (Button and Robertson, 2001). It should be noted, however, that such potential biases are inherent to any DNA-based strategy for relative quantification of microbial groups within a complex community, including quantitative PCR, phylogenetic microarrays as well as clone library analysis.

### Application of the HITChip for phylogenetic profiling and analysing the temporal dynamics of adult intestinal microbiota

The human gastrointestinal microbiota, similarly to all complex microbial ecosystems, is in a process of undergoing description. Each report of the diversity of this ecosystem based on SSU rRNA gene sequencing reveals a significant proportion of novel phylotypes (Eckburg *et al*., 2005; Ley *et al*., 2006; Leitch *et al*., 2007). Additionally, it has been found that about 70% of the human gastrointestinal microbiota phylotypes are subject-specific (Ley *et al*., 2006). Hence, the interpretation of data obtained with phylogenetic microarrays should go beyond the identification of known phylotypes. Although probes designed for the HITChip platform reported here and for other microarray platforms (Palmer *et al*., 2006) can be designed to target only the presently acknowledged diversity of intestinal microbiota, cross-hybridizations with related, not yet acknowledged members of the ecosystem still provide diagnostic information about the community composition (Gentry *et al*., 2006). Therefore, probe signal signatures can be used to obtain informative fingerprints of analysed samples. Subsequent assignment of each probe to a phylogenetic group upgrades the obtained hybridization signals to a phylogenetic fingerprint that provides information at any of the three defined levels of resolution (phylotype, genus-like, order).

**Fig. 3:**
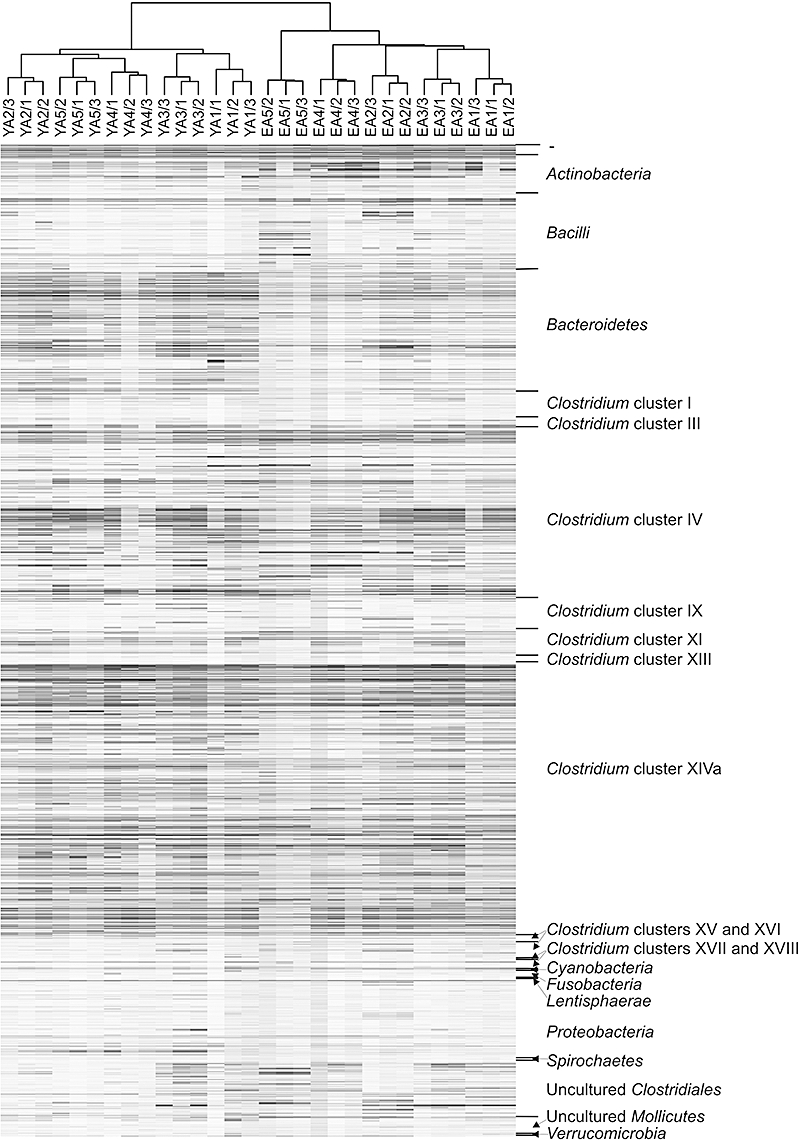
Clustering of the HITChip phylogenetic fingerprints of the human gastrointestinal microbiota of three faecal samples of ten subjects collected during period of at least two months. Samples are encoded by YA1–YA5 for younger adults, and by EA1–EA5 for elderly adults, while 1, 2 or 3 subsequent to slash sign indicate sample collection sequence. The highest phylogenetic level of specificity of probes (level 1) is depicted on the right panel of the figure.

To test the applicability of the HITChip platform as a phylogenetic fingerprinting tool, microbiota profiles were generated for 30 faecal samples collected at three time points over a period of at least two months from ten adult subjects, of which five elderly (average age 71 years) and five younger adults (average age 33 years). The faecal microbiota profiles of all samples were found to cluster in subject-specific triplicates (Fig. 3). The similarity of random pairs of the microbiota profiles of samples of a single individual was on average 92.2% (SD 3.0%). This was found to be higher (*P* < 0.001) than the similarity of the profiles of randomly selected samples from two different individuals, which was on average 72.0% (SD 9.2%). This result confirms previous observations that the adult faecal microbiota is individual-specific and relatively stable in time (Zoetendal *et al*., 1998; Matsuki *et al*., 2004; Ley *et al*., 2006). The level of similarity between faecal microbiota profiles of an individual computed using data obtained by HITChip analysis appeared to be higher than when fingerprints were generated using denaturing gradient gel electrophoresis (DGGE) of amplified 16S rRNA gene fragments (Zoetendal *et al*., 1998). Because of physical constraints (length of the gel), DGGE analysis generates a significantly lower number of datapoints as compared with a microarray-platform such as the HITChip. This suggests improved resolution of the analysis when phylogenetic microarrays are employed in comparison with the DGGE technique. Furthermore, the HITChip seems to be highly suitable for following the temporal dynamics of the intestinal microbiota as the hybridization signals of six probes per phylotype are relatively insensitive to the often observed temporal strain variation of the members of the microbiota (McCartney *et al*., 1996). This can be explained by the fact that a single nucleotide polymorphism, which is typical for the SSU rRNA gene sequence of different strains of a species (Klappenbach *et al*., 2001; Sacchi *et al*., 2005), would influence only the hybridization signal of one of the HITChip probes, while it would cause migration of the SSU rRNA gene segment of a phylotype onto a different position in a DGGE gel. Nevertheless, it should be noted that the outcome of the strain variation on a DGGE gel (i.e. shift of the band) will be the same as if one species was replaced by another from a different genus/family/class/order of even phylum.

The superiority of the HITChip over DGGE fingerprinting was further demonstrated in an experiment, where the long-term dynamics of the microbiota was analysed for five young adult individuals. The HITChip profiles consistently clustered per individual, whereas this was not observed for DGGE profiles of two of five individuals (Fig. S3). Another relevant advantage of phylogenetic microarray analysis over other fingerprinting techniques is the dramatically improved reproducibility of analysis and thus the reliability of results (Possemiers *et al*., 2004).

**Fig. 4:**
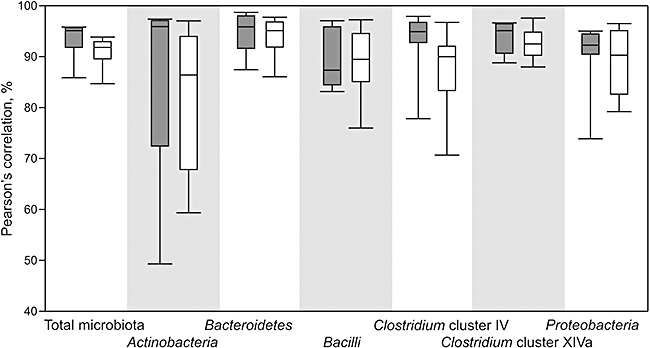
Similarity of the intestinal microbiota profiles for ten individuals calculated for 1 (grey bars) and two month (open bars) span. Results for total microbiota and six level 1 groups of the microbiota (phyla *Actinobacteria*, *Bacteroidetes* and *Proteobacteria*, and three groups within the *Firmicutes* phylum) are presented as box whisker plot. The box extends from the first quartile until the third quartile, with a line at the median, while whiskers are indicating minimal and maximal observation in the data set.

It has previously been shown using DGGE fingerprinting approaches that different members of the intestinal microbiota do not follow the same pattern of temporal change (Vanhoutte *et al*., 2004). Bearing this in mind, we have expanded the analysis of temporal variation of the total microbiota of ten adults for which time series of faecal samples were collected (see above). The variation in both young and elderly subjects at time spans of one and two months could be calculated for the total microbiota and different phylogenetic groups, namely the phyla *Actinobacteria*, *Bacteroidetes* and *Proteobacteria*, and three groups within the *Firmicutes* phylum (Fig. 4). The results show that the total microbiota, and all analysed phylogenetic groups, show higher similarity values for the 1 month time span than the 2 month time span. This reinforces previous hypotheses indicating that intestinal microbiota, although individual-specific, is influenced by environmental factors (Dethlefsen *et al*., 2006). Among different phylogenetic groups, members of the phylum *Actinobacteria* appeared to be affected the most by temporal variation, although pronounced changes in this microbial fraction appeared to be occurring exclusively in elderly subjects (75.4% for elderly versus 96.6% for young adults). Similarity values obtained for other phylogenetic groups for elderly and young adults were in good agreement, with the exception of *Clostridium* cluster IV after a time span of two months, during which this group changed notably more in young than in elderly adults (similarity values 82.9% and 91.4% respectively). The least affected by temporal variation in analysed subjects were the members of the phylum *Bacteroidetes* and *Clostridium* cluster XIVa, which are known to be the most abundant members of the intestinal microbiota (Zoetendal *et al*., 2004; Bäckhed *et al*., 2005; Eckburg *et al*., 2005).

Cluster analysis of the 30 microbiota profiles obtained by the HITChip analysis showed two distinct assemblies, each containing samples collected from five subjects (Fig. 3). The geographic origin of subjects recruited for this study (the Netherlands for young adults and Finland for the elderly subjects) could be contributing to the observed differences, but previous studies have failed to pinpoint difference in the microbiota of different Northern Europeans (Lay *et al*., 2005). Although other confounding effects, including differences in the DNA extraction procedures, cannot be excluded, it is more likely that observed differences are primarily caused by the different age of subjects, in line with previous observations that the microbiota of healthy middle-aged adults differs significantly from that of healthy elderly (Mueller *et al*., 2006). This was further supported by results obtained in a separate study, where DNA was extracted from faecal samples using two extraction methods that are essentially similar to these employed in this study, followed by the characterization of the resulting communities using Q-PCR and HITChip analysis (A. Salonen and W.M. de Vos, unpubl. obs.). It appeared that both extraction methods, although different in nature being mechanical and enzymatic, provided highly similar HITChip and other readings, with Pearsons coefficients of > 0.98 close to the reproducibility of the extraction (0.99), further reinforcing the notion that observed differences can be ascribed to the age rather than being a result of extraction bias. These compositional differences are even more pronounced when comparing adults and infants (Mitsuoka, 1992; Favier *et al*., 2002). The microbiota fingerprints generated using the HITChip are designed to indicate phylogenetic specificity of oligonucleotide probes and provide an instant visualization of the differences between the analysed samples. In this particular example, members of *Actinobacteria* and *Bacilli* could be identified as being more abundant in the microbiota of elderly subjects, while *Bacteroidetes* members were more dominant in the microbiota of young adults (Fig. 3). However, accurate identification of microbial groups that are marking the difference in the microbiota of two groups of subjects can be facilitated by semiquantitative interpretation of HITChip results, as shown in the following section.

### Quantitative differences between the microbiota of young and elderly adults

The potential of the HITChip to generate quantitative information on the human gastrointestinal microbiota was used for the identification of phylogenetic groups that are markedly different in relative abundance between the microbiota of young and elderly subjects. For a comprehensive analysis of the differences in the microbiota between young and elderly subjects, average hybridization signals for 129 genus-like phylogenetic groups were calculated for each sample. To appropriately explore this data set by multivariate statistical analysis, we have performed representational difference analysis in order to identify microbial groups of which the relative abundance is affected by the sample source (i.e. young versus elderly subjects) (ter Braak and Šmilauer, 2002; Lepš and Šmilauer, 2003). The overall microbiota composition appeared to be significantly different between groups of young and elderly subjects (*P* < 0.001). A more detailed analysis revealed that this difference could be mainly attributed to 20 out of 129 analysed phylogenetic groups (Table 2). The most pronounced differences could be observed for the order of *Bacilli*, of which several groups were found to have significantly higher abundance in elderly subjects. In contrast, a number of groups within the phylum *Bacteroidetes* were significantly more abundant in young adults.

**Table 2:** Level 2 (genus-like) groups targeted by the HITChip for which relative abundance was found to be significantly different between younger and elderly adults.

Phylum/order	Family/genus	Ratio elderly/younger	*P*-value
*Actinobacteria*	Actinomycetaceae	23	0.0012
	*Atopobium*	2	0.0135
*Bacilli*	*Lactobacillus salivarius* et rel.	10	0.0080
	*Aerococcus*	5	0.0087
	*Granulicatella*	15	0.0159
	*Streptococcus bovis* et rel.	5	0.0040
	*Streptococcus intermedius* et rel.	5	0.0081
*Clostridium* cluster XIVa	*Eubacterium hallii* et rel.	0.5	0.0416
*Clostridium* cluster XV	*Eubacterium limosum* et rel.	1.3	0.0144
*Bacteroidetes*	*Allistipes*	0.20	< 0.0001
	*Bacteroides ovatus* et rel.	0.14	0.0001
	*Bacteroides splachnicus* et rel.	0.14	< 0.0001
	*Bacteroides stercoris* et rel.	0.09	0.0017
	*Parabacteroides*	0.14	< 0.0001
	*Prevotella ruminicola* et rel.	0.25	0.0118
	Uncultured *Porphyromondaceae*	0.13	< 0.0001
	Uncultured *Bacteroidetes*	0.08	0.0005
*Betaproteobacteria*	*Aquabacterium*	0.13	0.0004
	*Burkholderia*	0.33	0.0395
*Gammaproteobacteria*	*Xanthomonadaceae*	0.06	0.0017

### Application of the HITChip reveals core microbiota in adults

**Fig. 5:**
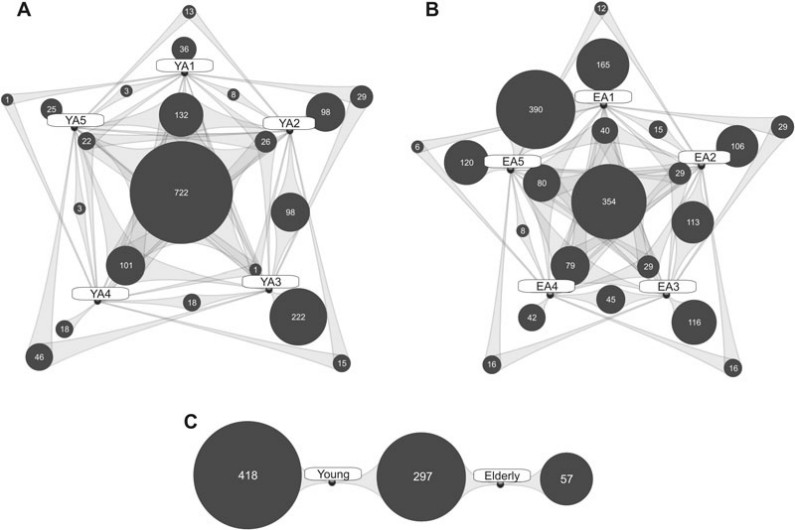
Venn diagram showing the distribution of the HITChip probes that showed significant hybridization signal when analysing five younger adults (A), five elderly adults (B) and between the core of younger and elderly adults (C). Only the core probes (those that were corresponding in all three analysed samples per subject) were taken into consideration for this analysis. For subject codes see Fig. 3. The image was generated using AutoFocus software (Aduna B.V., the Netherlands).

The observation that each individual harbours a unique faecal microbiota as reinforced by the present data (see above) has led to the question whether there exists microbial species that are present in all individuals, the so-called universal core that can be differentiated from the so-called individual core, consisting of species that are retained in a single individual over a certain period of time (Zoetendal *et al*., 2008). To further elaborate on this concept of a phylogenetic universal core we searched for those HITChip probes that were detected in all samples of an individual, and were shared between one or more individuals. This allowed us to construct Venn diagrams for the core probes for young and elderly adults (Fig. 5). In line with the lower interindividual similarity of the microbiota of elderly subjects, only 18.9% of positively hybridizing probes were found to respond in all samples of five analysed individuals. In contrast, the number of commonly responding probes was almost twofold higher in young adults when compared with the situation in the elderly. Despite these quantitative differences with respect to the core size, the overall phylogenetic composition of the microbiota core appeared to be similar in the young microbiota and elderly adults (Table S3). This analysis indicates that although the faecal samples of young and elderly adults contain different marked microbiota (Fig. 3), the different groups of bacteria have a highly similar phylogenetic position and may perform similar functions, lending support for the functional core hypothesis. In line with this, the analysis of microbiota in a large number of identical twin pairs indicated an above-species-level phylogenetic conservation, based on 16S rRNA gene sequences, which was linked to a functional core based on metagenome sequence analysis (Turnbaugh *et al*., 2009).

## Concluding remarks

In this paper, we present a straight-forward and flexible strategy for the systematic design of oligonucleotide probes that was applied to develop the HITChip platform, a phylogenetic microarray for studying human intestinal microbiota. This systematic probe design approach allows easy addition of probes that target newly discovered members of an ecosystem. This is highly valuable for presently insufficiently described ecosystems such as the human intestinal microbiota and allows to incorporate also partial 16S rRNA sequences such as derived from pyrosequencing (Andersson *et al*., 2008). Although the reliable prediction of the probe behaviour is hampered by the fact that not all relevant parameters that influence the probe–target duplex behaviour have yet been identified, microarrays based on the hypervariable regions of the SSU rRNA enable rapid, high-resolution fingerprinting of human gastrointestinal samples. With the possibility for relative quantification, which allows for simultaneous comparison of the relative amounts of over hundred genus-like groups of intestinal bacteria, the HITChip has a great potential to provide better insight into the human gastrointestinal microbiota, to identify the effect of time or dietary changes and to compare groups of individuals that differ in the genotype or health status, age, geographic origin or any other relevant factor, potentially involved in shaping of this tremendously important ecosystem. A first application of the HITChip in comparing young and adult microbiota showed notable differences and provided new insight in the effect of aging on the human intestinal microbiota.

## Experimental procedures

### *In silico* analysis of SSU rRNA gene sequences

The SSU rRNA gene sequences of the human gastrointestinal microbiota were extracted from the ARB database release 2002 (Ludwig *et al*., 2004), amended with sequences of newly reported isolates and sequences amplified from gastrointestinal samples. The updated ARB database therefore contained most of the human intestinal microbiota detected in both cultivation-dependent and -independent studies reported until December 2006. Over 16 000 sequences, for the majority derived from healthy individuals from Europe, Asia and USA, were organized into 1140 distinct phylotypes, defined as monophyletic groups of sequences with less than 2% sequence divergence. Furthermore, the database was upgraded by assigning each of the human gastrointestinal phylotypes to their phylogenetic position. However, the use of nomenclatural taxonomy is hampered by the fact that many SSU rRNA sequences obtained from the human gastrointestinal samples represent uncultured organisms that are only distantly related to any cultured representative, and therefore their taxonomic position cannot be accurately determined. Therefore, the sequences were organized into alternative SSU rRNA-based phylogenetic groups similar to those previously proposed for the genus *Clostridium* (Collins *et al*., 1994). Three levels of phylogeny were defined: (i) level 1 corresponds to the phylum, or in case of Firmicutes to the *Clostridium* cluster, (ii) level 2 includes groups of sequences with 90% or more sequence similarity and (iii) level 3 represents unique phylotypes that were defined as species for cultivated microorganisms, or representatives of each monophyletic group with = 98% sequence identity for clones corresponding to uncultured microorganisms (Table S3). Nine variable regions of the SSU rRNA gene were exported for 1140 sequences representative of prokaryotic gastrointestinal phylotypes. The sequences were exported using appropriate bordering nucleotide positions (Table 1). These were determined based on the alignment positions of primers available for the amplification of each of the variable regions. Unique SSU rRNA sequences were identified using Pivot Tables built from the exported sequence data in Microsoft Excel.

### Probe design

Antisense oligonucleotide probes were designed based on the reverse complement of two variable regions that were selected based on their low redundancy. Reverse complements of the SSU rRNA gene sequences were produced in ARB prior to export. Variable regions were split into three overlapping probes of 24 nt with the use of available Microsoft Excel functions. The theoretical melting temperature (Tm) of probe-perfect match target duplexes was predicted using an earlier reported equation (Sambrook *et al*., 1989a), assuming a sodium ion concentration of 0.5 M. The probe length was adjusted by extending or restricting the overlap between probes until the corresponding Tm fitted a narrow range of 70 ± 2°C. The size of the probes was kept between 18 and 30 nt to enable similar duplex kinetics. Following this probe design procedure, six probes were designed per each complete SSU rRNA gene sequence type. The prediction of Tm was re-evaluated using the nearest neighbour Tm prediction algorithm with previously proposed parameters (SantaLucia, 1998), as it has been observed that the outcome of different Tm prediction algorithms can be notably different (Panjkovich and Melo, 2005). The predicted Tm of 95% of probes fitted into the narrow range of ± 3.5°C, as assessed using the SantaLucia algorithm, which is in the range of prediction error of this algorithm (Panjkovich and Melo, 2004), and comparable to the initially predicted range of melting temperatures.

### Preparation of artificial mixtures

Artificial mixes were prepared by combination of human gastrointestinal phylotypes obtained in two SSU rRNA clone libraries previously prepared from human gastrointestinal samples (Zoetendal *et al*., 1998; Suau *et al*., 1999). SSU rRNA gene inserts were amplified by the use of appropriate vector-targeting primers using the GO Taq PCR amplification kit (Promega, Leiden, the Netherlands). PCR was performed on 1 μl of cell-lysates (containing approximately 10 ng of plasmid DNA) from clones using a set of Sp6 and T7 primers for clones obtained by Zoetendal and colleagues (1998) and the pUAg primer set for clones obtained by Suau and colleagues (1999).

### Faecal DNA extraction

Faecal samples used in this study were collected as part of two independent trials, results of which are reported separately (Lay *et al*., 2005; Surakka *et al*., 2009). Total DNA from faecal material collected from younger adults was extracted using a modified protocol of the FastDNA SPIN Kit for Soil (Qbiogene, Basel, Switzerland) (Zoetendal *et al*., 2006b), while DNA from faecal material collected from elderly subjects was extracted according to the protocol described by Myllyluoma and colleagues (2005). DNA yield was quantified using a NanoDrop ND-1000 spectrophotometer (NanoDrop Technologies, Wilmington, DE). DNA concentration was adjusted to 10 ng ml^−1^ and was used as a template for PCR amplification.

### RNA preparation

The SSU rRNA gene was re-amplified from plasmid clone inserts or amplified from faecal DNA using the primers *T7prom*-Bact-27-for (5′-TGA ATT GTA ATA C GA CTC ACT ATA GGG GTT TGA TCC TGG CTC AG-3′) and Uni-1492-rev (5′-CGG CTA CCT TGT TAC GAC-3′), which ensured the introduction of a T7 promoter sequence at the 5′ terminus of the rRNA gene amplicon. PCR reactions were carried out in a final volume of 50 μl, and 10 ng of DNA samples was used as template. Samples were initially denatured at 94°C for 2 min followed by 35 cycles of 94°C (30 s), 52°C (40 s), 72°C (90 s) and a final extension at 72°C for 7 min. The PCR products were purified using the DNA Clean and Concentrator kit (Zymo Research, Orange, USA), according to the manufacturer's instruction. Final DNA concentration was determined using a NanoDrop spectrophotometer as described above.

*In vitro* transcription of T7-promoter-carrying SSU rRNA gene was performed according to the manufacturer's protocol using the Riboprobe System (Promega, La Jolla, USA), 500 ng of the T7-16S rRNA gene PCR-product and, besides rATP, rGTP, rCTP, a 1:1 mix of rUTP and aminoallyl-rUTP (Ambion, Austin, Tx, USA). The transcription reaction was performed at room temperature for 2 h, the template DNA was digested applying the Qiagen RNAse-free DNAse kit (Qiagen, Hilden, Germany) and RNA was purified using the RNeasy Mini-Elute Kit (Qiagen, Hilden, Germany). RNA yield was measured as described above.

Amino-allyl-modified nucleotides were coupled with CyDye using the Post-Labelling Reactive Dye (Amersham Biosciences, Little Chalfont, UK), previously dissolved in 84 μl dimethyl sulfoxide. Labelling reactions were performed in a 25 mM sodium bicarbonate buffer (pH 8.7) by adding 20 μl of dissolved CyDye to 2 μg of purified RNA in a final volume of 40 μl. Samples were incubated for 90 min in the dark at room temperature. The reaction was stopped by adding 15 μl of 4 M hydroxyl-amine and incubating for 15 min in the dark. RNAse-free water was added to 100 μl and labelled RNA was purified and quantified as described above.

### Microarray production and hybridization

Microarrays were custom-synthesized by Agilent Technologies (Agilent Technologies, Palo Alto, CA). The oligonucleotide probes were extended at the 3′ end (at the array support side) by 10-nt-long T spacers and were printed on the array using *in situ* surface-attached oligonucleotide probe synthesis (Blanchard *et al*., 1996). Arrays used in this study were of the 2 × 11K format, with two arrays per glass slide. Each array was hybridized with two samples, labelled with Cy3 and Cy5 respectively. Combined Cy3- and Cy5-labelled target mixtures were fragmented by adding 1 μl of Ambion 10× fragmentation reagent (Ambion), and incubation for 20 min at 70°C, according to the manufacturer's instruction. Fragmentation was stopped by adding 1 μl of Ambion stop solution. Hybridization mix was prepared by adding to the RNA mixture 31.5 μl of 20× SSC (Sambrook *et al*., 1989b), 6.3 μl of 10% SDS (Sambrook *et al*., 1989b), 25 μl of Agilent Control Target mix and RNAse-free water to a total volume of 210 μl. Hybridization was carried out at 62.5°C in a rotation oven (Agilent) for 16 h. Slides were washed at room temperature in 2× SSC, 0.3% SDS for 10 min and at 38°C in 0.1× SSC, 0.3% SDS for 10 min SDS was completely removed by washing the slides in 0.06× SSPE (Sambrook *et al*., 1989b) for 5 min followed by a quick dry with compressed air.

### Support database construction

The data were stored in a custom-designed relational database, which runs under the MySQL database management system (http://www.mysql.com/). The database design was divided into sections covering sample annotation, microarray raw and normalized data, microarray design, probe characteristics, probe–target pairs and the phylogenetic position of the SSU rRNA target molecules. Using the sequences of the SSU rRNA molecules stored in the database that, in addition to unique phylotypes, contained sequences with more than 98% sequence similarity to one of the unique phylotypes (total 2681 sequences), putative probe–target pairs were selected using a standalone version of the blast program from NCBI (Altschul *et al*., 1990) with the following parameter settings: gapopen = 3, gapextend = 2, matchreward = 1, mismatchpenalty = −1, wordsize = 7, and expect = 1. A perfect-match Tm of each probe–target pair on the microarray was calculated assuming sodium ion concentration of 0.5 M and a nucleotide concentration of 10^−12^ M using the nearest neighbour algorithm (SantaLucia, 1998). In this probe–target pair hybrid calculation the whole probe, including the poly T spacer, was taken into account, leaving open the possibility that some nucleotides from the spacer take part in the hybrid pair. The nearest neighbour algorithm was also used to calculate the perfect-match Tm for a perfectly matching target for each probe, excluding the poly T spacer region. Raw microarray data files from the feature extraction software as well as tab-delimited files containing information about the sample annotations were imported into the database by converting them to SQL scripts that were subsequently executed.

### Microarray data extraction and analysis

Data were extracted from microarray images using the Agilent Feature Extraction software, versions 7.5-9.1 (http://www.agilent.com). Normalization of the microarray data was performed in three steps: spatial normalization, outlier detection and sample-wise quantile normalization.

Spatial normalization was carried out for each channel separately to correct spatial defects in the arrays. Spatial defects were especially clear in a number of arrays in the lower signal intensity ranges. Therefore, the 70% lower quantile of foreground signals were fitted to a two-dimensional polynomial surface using an implementation written by B.D. Ripley in R statistical software (http://www.r-project.org/) of the loess algorithm (Cleveland *et al*., 1992). A spatially normalized signal for each spot was calculated by subtracting the local loess predicted signal from the raw foreground signal and adding the global minimum of the fitted polynomial surface. The resulting signals were stored as the spatially normalized signal in the database.

Outlier measurements in the spatially normalized signals were detected and flagged automatically. As each sample was labelled with both dyes and hybridized to two different arrays printed on different slides, and as each probe was spotted at least twice on the array, at least four replicate measurements per probe and sample were available. Outliers in these replicate measurements were detected using an implementation in the ‘outlier’ package in R of a chi-square test proposed by Dixon (Dixon, 1950). The test was applied to the logarithmically transformed signals, and the average over the whole probe set of the variances per probe was substituted in the variance parameter for this test. The significance level of outlier detection was set at 0.001.

The reproducibility of the hybridization was assessed by calculating the Pearson's linear correlation of the natural logarithm of spatially normalized signals excluding outlier spots (Tan *et al*., 2003). A correlation coefficient of minimally 0.98 was considered to indicate a satisfactory reproducibility. Further normalization was performed using only arrays that contained data of satisfactory correlation.

A quantile normalization was applied to the collective spatially normalized measurements of each sample (Bolstad *et al*., 2003). The rationale assumption behind this normalization is that the distribution of signals should be the same for each measurement of a sample. The sample-wise quantile normalized signals were also stored in the database and used for further analyses.

### Calculation of probe profiles

For each probe, the specific target SSU rRNA sequences in the database were identified by selecting those targets for which the Tm was equal to or higher than the perfect-match Tm of the probe. The occasional occurrence of Tm's higher than those observed for perfect match target sequences was due to the fact that a nucleotide of the poly T spacer, which was taken into account for Tm calculation, makes a perfect additional match with the target. The phylogenetic position of these targets was scored based on the ARB database assigned clustering of human gastrointestinal sequences described above. The lowest phylogenetic level to which all theoretically hybridizing targets belonged was recorded as the specific phylogenetic level of the probe.

Hierarchical clustering of probe profiles was carried out by calculating a distance matrix between the samples based on the squared difference between each pair of profiles (Euclidian distance). The distance matrix was used in the hclust implementation in R of a hierarchical clustering algorithm. The agglomeration method used in this algorithm was Ward's minimum variance method.

Similarity of the total microbiota composition based on the HITChip profiles was assessed by calculating Pearson's product moment correlation (Pearson's correlation) that reflects the degree of linear relationship between analysed data sets. In addition to the assessment of the total microbiota similarity, Pearson's correlation was calculated for sets of probes that correspond to different phylogenetic groups. Strong positive association (ρ = 0.999) was obtained by comparison of Pearson's correlation calculated for total HITChip profiles obtained from *in vitro* transcribed *Bifidobacterium*-specific PCR products with Pearson's correlation calculated for *Bifidobacterium*-specific probes for five pairs of samples (Fig. S4). This correlation could not be assessed for other relevant gastrointestinal groups as the group-specific primers do not allow amplification of the SSU rRNA segment that is used for the HITChip analysis and is between *Escherichia coli* positions 64–1047 (Matsuki *et al*., 2002; Fogarty and Voytek, 2005; Shen *et al*., 2006).

The cut-off value for positively responding probes was defined as a probe intensity value at which the first differential of the function fitted to all probes sorted by signal intensities was equal to 1, which represents the point where the slope of the fitted curve changes. The cut-off value could be approximated as the probe signal intensity that is 25% higher than the average value of signal intensity of 500 probes with the weakest signal.

### Statistical analysis

To evaluate if two data sets were significantly different, a *P*-value was calculated using Student *t*-test for normally distributed data assuming equal variables and two tailed distribution. Wilcox rank sum test (Bauer, 1972) was applied for data that significantly deviated from normal distribution (*P*-value 0.05). Normality of data sets was tested using Shapiro–Wilk test (Royston, 1982). These tests were performed using R statistical software (http://www.r-project.org/). If statistical tests were performed on a large number of variables, *P*-values were Bonferroni-corrected.

## Supporting information

**Fig. S1.** Relative change of the hybridization signal of phylotype-specific HITChip probes with the change of the relative abundance in an artificial target mix of 40 phylotypes, assessed for varying concentrations of 10 gastrointestinal phylotypes. Average data with the respective standard error are presented.

**Fig. S2.** Influence of the PCR cycle number on reproducibility of HITChip fingerprint. Clustering of the HITChip fingerprints is shown for two faecal samples of five young adult subjects collected during a period of 2 months. Samples are encoded by YA1-YA5, while 1 or 2 subsequent to slash sign indicate sample collection sequence. The letter b indicates that fingerprints were obtained with 20 cycles of 35 PCR, whereas for all other profiles, 35 cycles were used. The highest phylogenetic level of specificity of probes (level 1) is depicted on the right panel of the figure.

**Fig. S3.** Fingerprints of the human faecal microbiota obtained using DGGE analysis (a) and the HITChip (b) of five subjects which are coded V1-V5. Two samples per subject were analysed, and samples were collected with a time span of 4 years, where the numbers subsequent to slash sign indicate the time span in years after the first sampling. The highest phylogenetic level of specificity of the HITChip probes is depicted on the right panel of the figure.

**Fig. S4.** Similarity between the *Bifidobacterium* communities in the faecal microbiota of five pairs of monozygotic twins calculated as Pearson's correlation. The similarity was assessed by comparing total hybridization profiles obtained for *in vitro* transcribed *Bifidobacterium*-specific PCR product (*y*-axis) to hybridization signals of *Bifidobacterium*-specific probes obtained with *in vitro* transcribed universal bacterial PCR product (*x*-axis).

**Table S1.** Classification of phylotypes found in the human intestine based on the SSU rRNA gene similarity. Level 1 corresponds to the phylum, or in case of *Firmicutes* to the *Clostridium* cluster; level 2 includes groups of sequences with 90% or more sequence similarity; level 3 represents unique phylotypes that were defined as species for cultivated microorganisms, or representatives of each monophyletic group with ≥ 98% sequence identity for clones corresponding to uncultured microorganisms.

**Table S2.** Pairs of human gastrointestinal species and yet uncultured phylotypes that cannot be distinguished based on their variable region V1 and V6 SSU rRNA gene sequence.

**Table S3.** Phylogenetic identification of core probes.
