# Supplementary figures and images for: Development and application of the human intestinal tract chip, a phylogenetic microarray: analysis of universally conserved phylotypes in the abundant microbiota of young and elderly adults
Source: Environ Microbiol. 2009 Jul;11(7):1736–51. doi: 10.1111/j.1462-2920.2009.01900.x (PMC2784037; doi:10.1111/j.1462-2920.2009.01900.x)

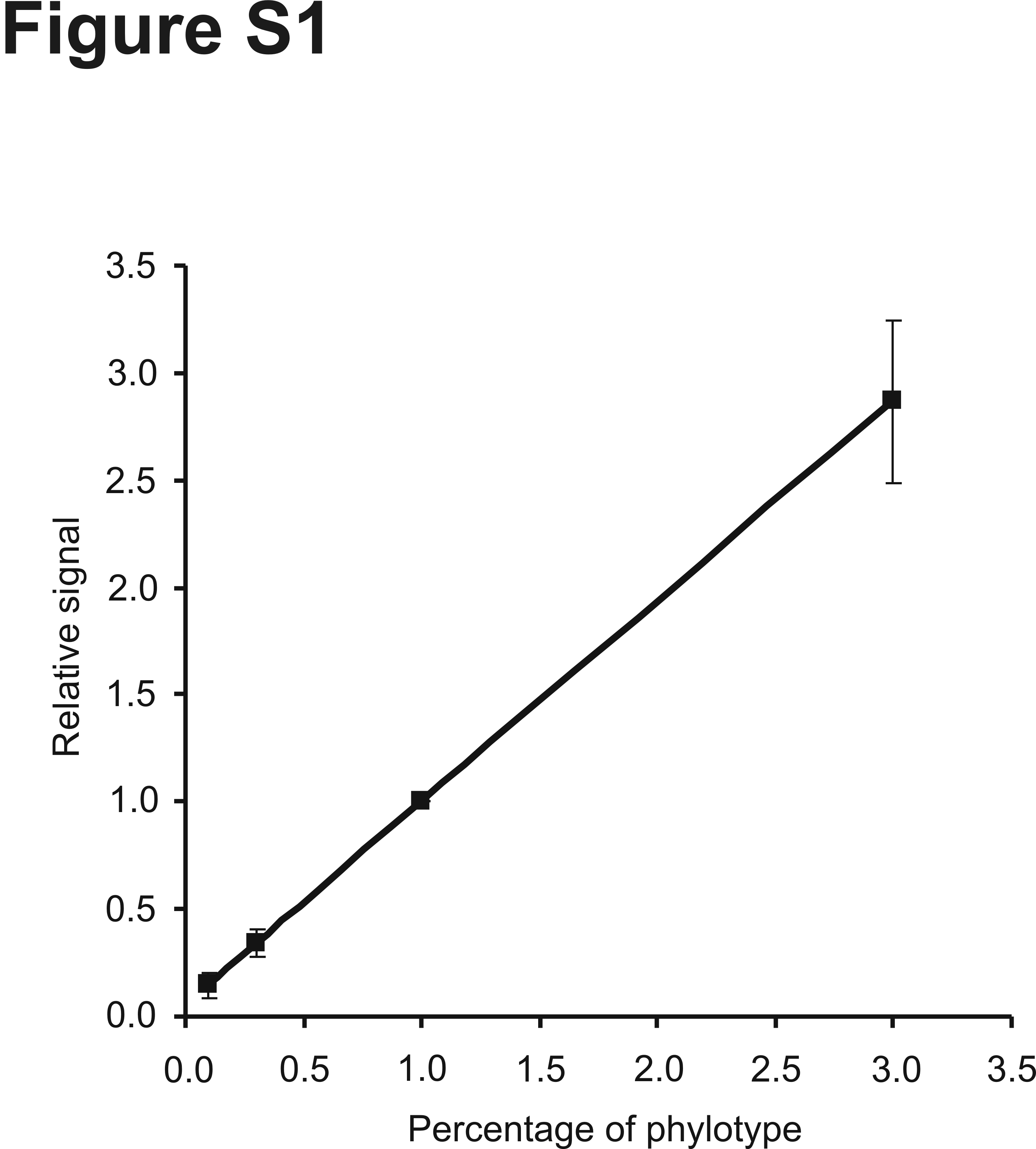

Supplement: Supplementary file 1 [file emi0011-1736-SD1.tif]

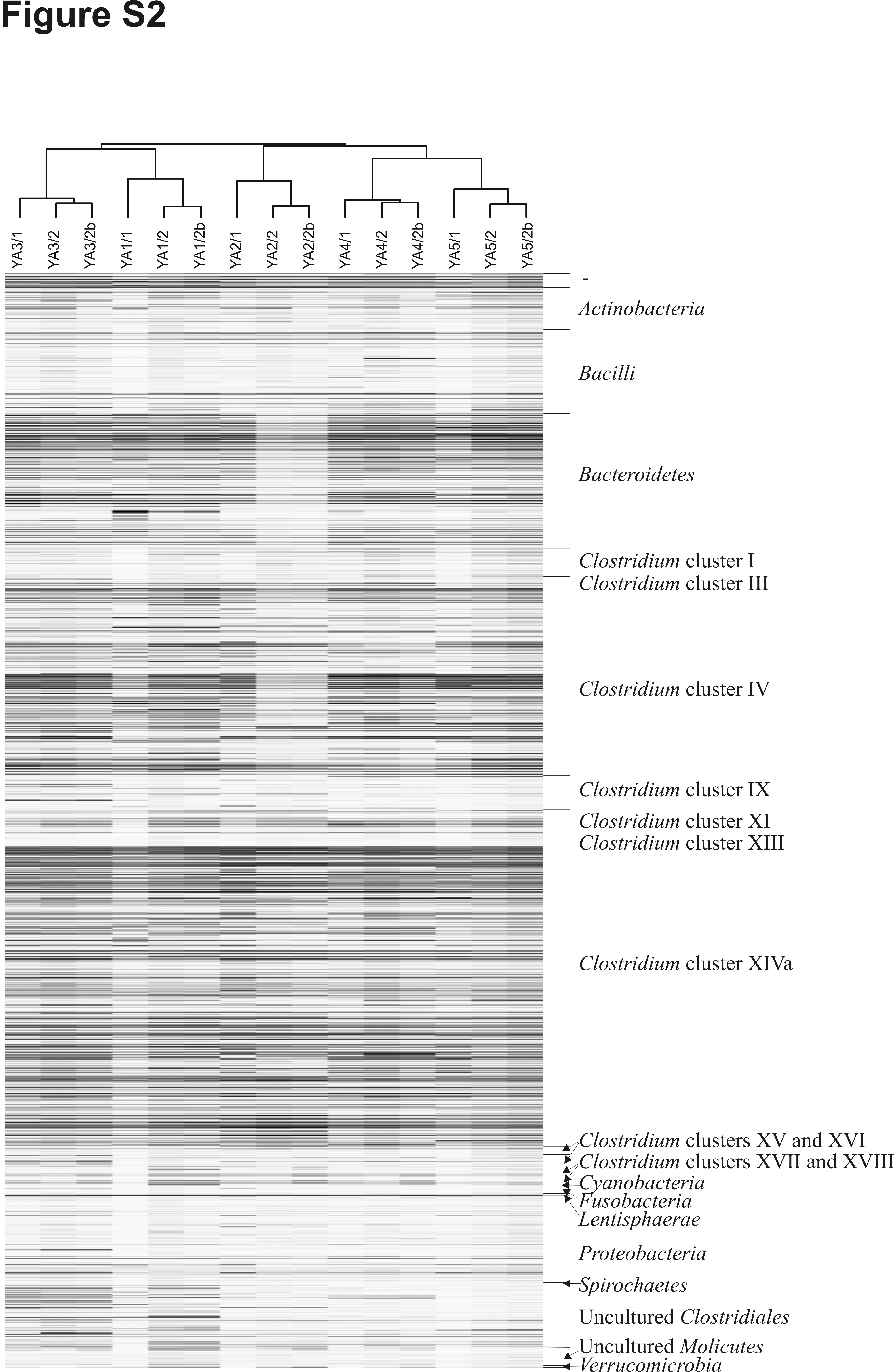

Supplement: Supplementary file 2 [file emi0011-1736-SD2.tif]

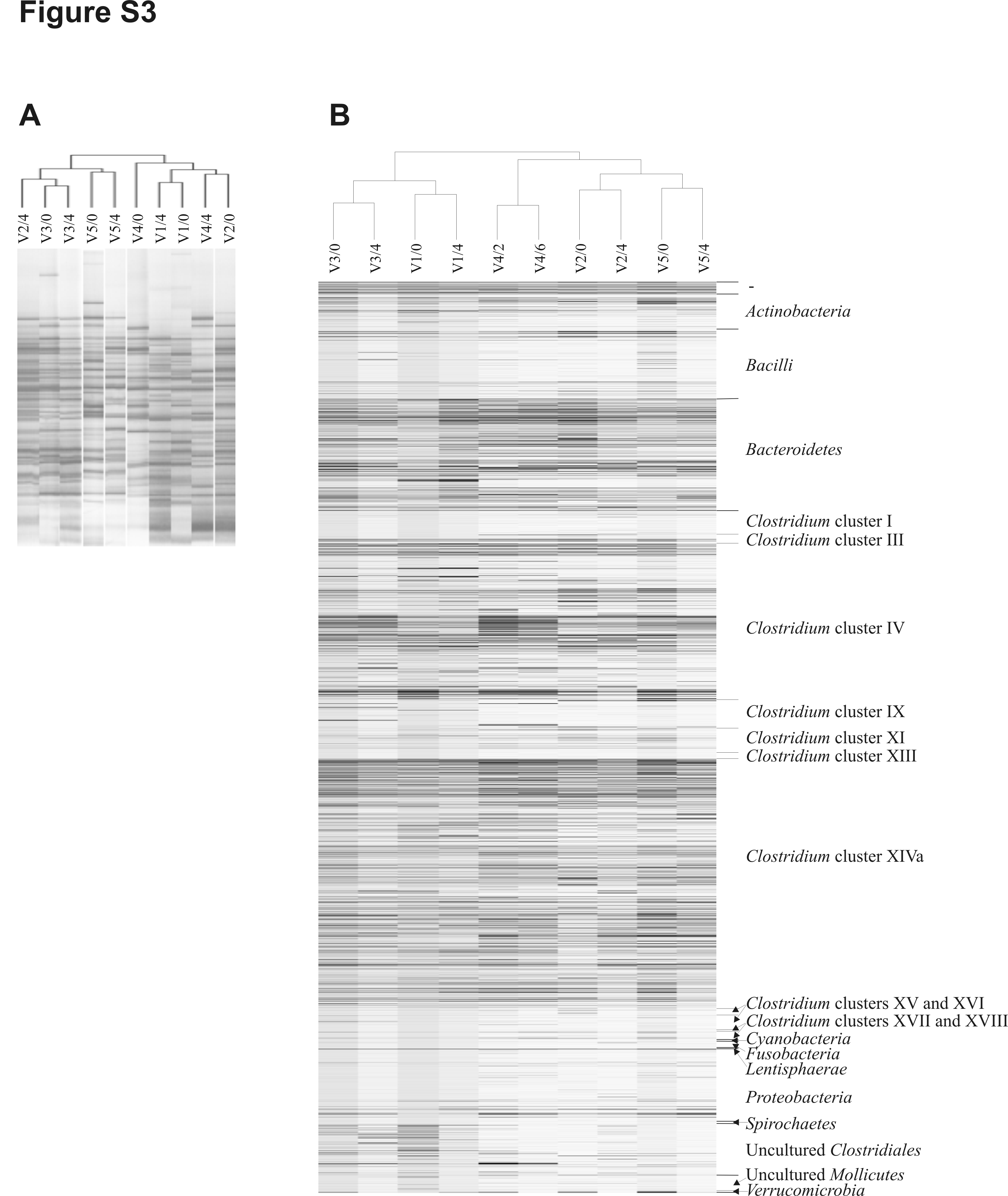

Supplement: Supplementary file 3 [file emi0011-1736-SD3.tif]

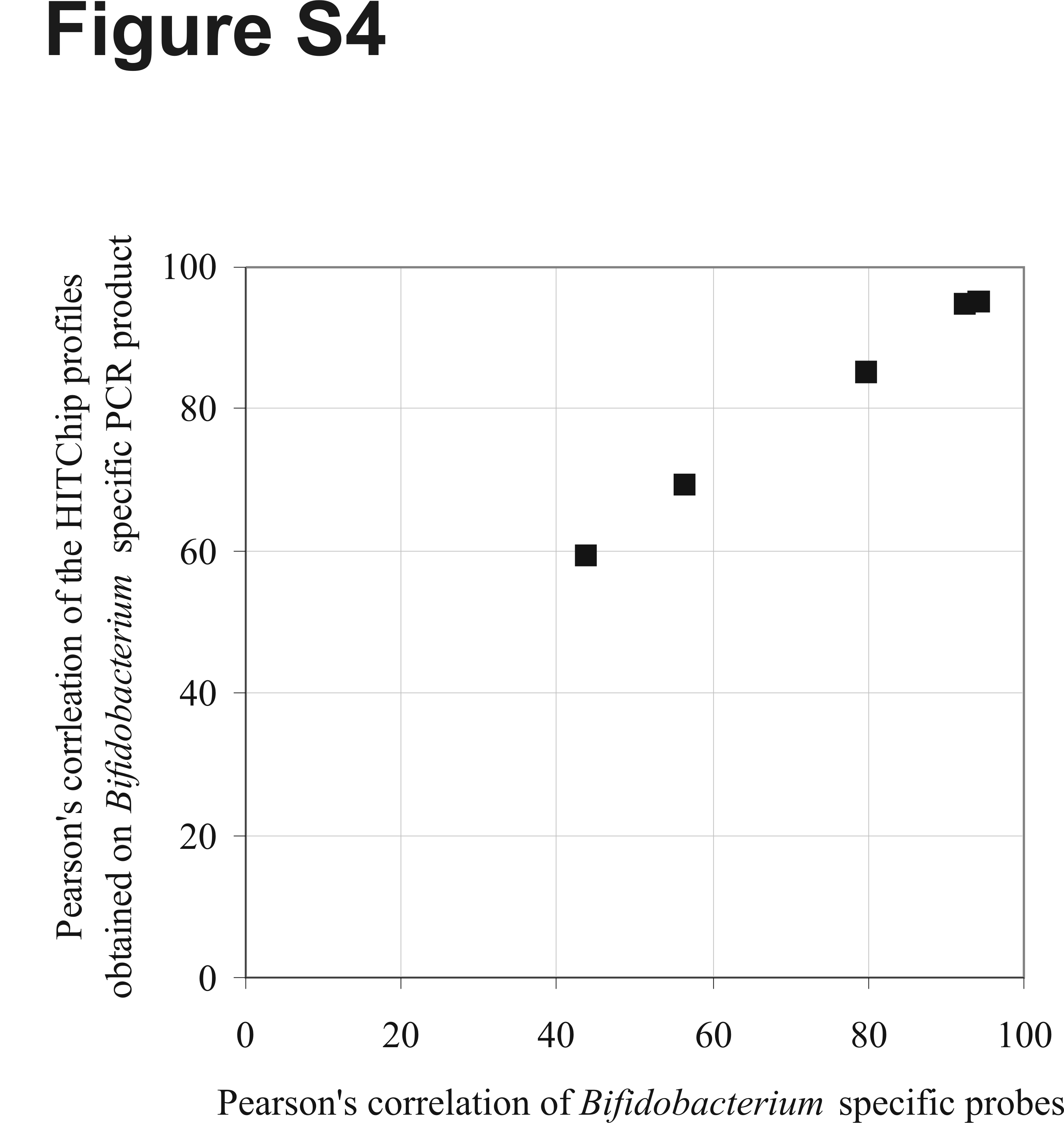

Supplement: Supplementary file 4 [file emi0011-1736-SD4.tif]
