# Supplementary material for: Development and application of the human intestinal tract chip, a phylogenetic microarray: analysis of universally conserved phylotypes in the abundant microbiota of young and elderly adults
Source: Environ Microbiol. 2009 Jul;11(7):1736–51. doi: 10.1111/j.1462-2920.2009.01900.x (PMC2784037; doi:10.1111/j.1462-2920.2009.01900.x)
Supplement: Supplementary file 5 [file emi0011-1736-SD5.doc]

**Table S1** Classification of phylotypes found in the human intestine based on the SSU rRNA gene similarity. Level 1 corresponds to the phylum, or in case of *Firmicutes* to the *Clostridium* cluster; Level 2 includes groups of sequences with 90% or more sequence similarity; Level 3 represents unique phylotypes that were defined as species for cultivated microorganisms, or representatives of each monophyletic group with ≥ 98% sequence identity for clones corresponding to uncultured microorganisms.

| Level 1 | Level 2 | Level 3 | Accession number |
| --- | --- | --- | --- |
| *Actinobacteria* | *Actinomycetaceae* | *Arcanobacterium pyogenes* | M29552 |
|  |  | *Actinomyces naeslundii* | M33911 |
|  |  | Uncultured bacterium clone Eldhufec234 | AY920109 |
|  |  | Uncultured bacterium clone Eldhufec081 | AY919956 |
|  |  | uncultured bacterium Z650 | AY979340 |
|  |  | uncultured bacterium NH01 | AY978941 |
|  | *Atopobium* | *Atopobium parvulum* | AF292372 |
|  |  | *Atopobium minutum* | M59059 |
|  | *Bifidobacterium* | *Bifidobacterium breve* | AB006658 |
|  |  | *Bifidobacterium thermophilum* | AB016246 |
|  |  | *Bifidobacterium angulatum* | D86182 |
|  |  | *Bifidobacterium dentium* | D86183 |
|  |  | *Bifidobacterium infantis* | D86184 |
|  |  | *Bifidobacterium pseudocatenulatum* | D86187 |
|  |  | *Bifidobacterium gallicum* | D86189 |
|  |  | *Bifidobacterium pseudolongum* | D86194 |
|  |  | *Bifidobacterium bifidum* | M38018 |
|  |  | *Bifidobacterium adolescentis* | M58729 |
|  |  | *Bifidobacterium catenulatum* | M58732 |
|  |  | *Bifidobacterium longum* | M58739 |
|  |  | *Bifidobacterium* sp. CB8 | AB064925 |
|  |  | Uncultured bacterium clone Eldhufec082 | AY919957 |
|  |  | uncultured bacterium (human infant) L14E | AF253371 |
|  |  | uncultured bacterium (human infant) N14A | AF253397 |
|  |  | uncultured bacterium Adhufec069rbh | AY471706 |
|  |  | uncultured *Bifidobacterium* sp. 15D | AF275886 |
|  |  | uncultured *Bifidobacterium* sp. 13D | AF275884 |
|  |  | *Bifidobacterium* sp. PL1 | AF306789 |
|  | *Collinsella* | *Collinsella aerofaciens* | AB011814 |
|  |  | *Collinsella* sp. CB52 | AB064936 |
|  |  | Uncultured bacterium clone Eldhufec074 | AY919949 |
|  |  | *Collinsella stercoris* | AB031062 |
|  |  | *Collinsella intestinalis* | AB031063 |
|  | *Corynebacterium* | *Corynebacterium xerosis* | AF024653 |
|  |  | *Corynebacterium ulcerans* | X81911 |
|  |  | *Corynebacterium ammoniagenes* | X82056 |
|  |  | *Corynebacterium pseudodiphtheriticum* | X84258 |
|  |  | uncultured bacterium LI92 | AY978122 |
|  |  | uncultured bacterium N337 | AY980429 |
|  | *Eggerthella lenta et rel.* | *Eggerthella lenta* | AB011817 |
|  |  | uncultured Gram-positive bacterium NO1H5 | AB064862 |
|  |  | uncultured bacterium ME67 | AY916234 |
|  |  | Uncultured bacterium clone Eldhufec078 | AY919953 |
|  |  | Uncultured bacterium clone Eldhufec076 | AY919951 |
|  |  | Uncultured bacterium clone Eldhufec075 | AY919950 |
|  |  | *Denitrobacterium* sp. CCUG 45665 | AJ518870 |
|  |  | uncultured bacterium Adhufec036abh | AY471677 |
|  | *Micrococcaceae* | *Micrococcus luteus* | AJ276811 |
|  |  | *Rothia dentocariosa* | M59055 |
|  |  | Uncultured bacterium clone Eldhufec080 | AY919955 |
|  |  | uncultured bacterium HuJJ72 | AY684419 |
|  | *Propionibacterium* | *Propionibacterium acnes* | AB041617 |
|  |  | *Propionibacterium avidum* | AJ003055 |
|  |  | *Propionibacterium granulosum* | AJ003057 |
|  |  | *Propionibacterium propionicum* | X53216 |
|  |  | *Propionibacterium jensenii* | X53219 |
|  |  | *Propionibacterium acidipropionici* | X53221 |
| *Bacteroidetes* | *Alistipes et rel.* | *Alistipes putredinis* | L16497 |
|  |  | *Bacteroides* sp. CJ44 | AB080886 |
|  |  | uncultured bacterium C706 | AY916343 |
|  |  | uncultured bacterium D080 | AY916354 |
|  |  | uncultured bacterium M162 | AY916149 |
|  |  | uncultured bacterium MG06 | AY916286 |
|  |  | uncultured bacterium NH37 | AY916174 |
|  |  | uncultured bacterium NN46 | AY916247 |
|  |  | Uncultured bacterium clone Eldhufec050 | AY919925 |
|  |  | Uncultured bacterium clone Eldhufec022 | AY919897 |
|  |  | uncultured bacterium cadhufec076h7 | AF530308 |
|  |  | uncultured bacterium adhufec52.25 | AF153864 |
|  |  | *Alistipes finegoldii* | AJ518874 |
|  |  | *Bacteroides* sp. DSM 12148 | AJ518876 |
|  |  | uncultured bacterium Adhufec002rbh | AY471693 |
|  |  | *Alistipes oderdonkii* | AY974072 |
|  |  | *Alistipes shahii* | AY974071 |
|  | *Bacteroides fragilis et rel.* | bacterium adhufec23 | AF132251 |
|  |  | bacterium adhufec355 | AF132263 |
|  |  | *Bacteroides thetaiotaomicron* | L16489 |
|  |  | *Bacteroides fragilis* | M11656 |
|  |  | uncultured bacterium MR34 | AY916210 |
|  |  | uncultured bacterium Z091 | AY916178 |
|  |  | Uncultured bacterium clone Eldhufec021 | AY919896 |
|  |  | uncultured bacterium LCRC79 | AF499852 |
|  |  | *Bacteroides finegoldii* | AB222699 |
|  |  | *Bacteroides nordii* | AY608697 |
|  |  | *Bacteroides salyersiae* | AY608696 |
|  | *Bacteroides intestinalis et rel.* | uncultured bacterium OLDA-A11 | AB099761 |
|  |  | uncultured bacterium HuCA21 | AJ409009 |
|  |  | *Bacteroides intestinalis* | AB214329 |
|  | *Bacteroides ovatus et rel.* | *Bacteroides ovatus* | L16484 |
|  |  | *Bacteroides caccae* | X83951 |
|  |  | uncultured bacterium NC94 | AY916170 |
|  |  | uncultured bacterium NP35 | AY916253 |
|  |  | uncultured bacterium HuCA34 | AJ408982 |
|  |  | uncultured bacterium HuCC30 | AJ315484 |
|  |  | Uncultured bacterium clone Eldhufec030 | AY919905 |
|  | *Bacteroides plebeius et rel.* | bacterium adhufec367 | AF132266 |
|  |  | *Bacteroides* sp. CO11 | AB064922 |
|  |  | uncultured bacterium D790 | AY916390 |
|  |  | Uncultured bacterium clone Eldhufec045 | AY919920 |
|  |  | Uncultured bacterium clone Eldhufec335 | AY920210 |
|  |  | *Bacteroides coprocola* | AB200225 |
|  |  | *Bacteroides plebeius* | AB200222 |
|  |  | uncultured bacterium Adhufec025abh | AY471674 |
|  |  | uncultured bacterium Adhufec086rbh | AY471710 |
|  | *Bacteroides splachnicus et rel.* | bacterium adhufec84 | AF132281 |
|  |  | *Bacteroides splanchnicus* | L16496 |
|  |  | uncultured bacterium C268 | AY916330 |
|  |  | uncultured bacterium MO48 | AY916145 |
|  |  | uncultured bacterium MN96 | AY916307 |
|  |  | uncultured bacterium NK71 | AY916241 |
|  |  | uncultured bacterium NK90 | AY916243 |
|  |  | uncultured bacterium NN42 | AY916246 |
|  |  | uncultured bacterium NN84 | AY916248 |
|  |  | uncultured bacterium NP53 | AY916254 |
|  |  | uncultured bacterium NX93 | AY916310 |
|  |  | Uncultured bacterium clone Eldhufec044 | AY919919 |
|  |  | Uncultured bacterium clone Eldhufec048 | AY919923 |
|  | *Bacteroides stercoris et rel.* | bacterium adhufec303 | AF132259 |
|  |  | *Bacteroides eggerthii* | L16485 |
|  |  | *Bacteroides stercoris* | X83953 |
|  |  | Uncultured bacterium clone Eldhufec057 | AY919932 |
|  |  | Uncultured bacterium clone Eldhufec025 | AY919900 |
|  | *Bacteroides uniformis et rel.* | *Bacteroides uniformis* | L16486 |
|  |  | uncultured *Bacteroides* sp. NS2A11 | AB064816 |
|  | *Bacteroides vulgatus et rel.* | *Bacteroides vulgatus* | M58762 |
|  |  | *Bacteroides dorei* | AB242142 |
|  | *Parabacteroides distasonis et rel.* | *Parabacteroides distasonis* | M25249 |
|  |  | *Parabacteroides merdae* | X83954 |
|  |  | uncultured bacterium OLDA-B10 | AB099754 |
|  |  | uncultured bacterium M270 | AY916152 |
|  |  | uncultured bacterium MH76 | AY916297 |
|  |  | Uncultured bacterium clone Eldhufec042 | AY919917 |
|  |  | uncultured bacterium LCLC20 | AF499837 |
|  |  | uncultured bacterium ABLCf15 | AF499899 |
|  |  | *Parabacteroides goldsteinii* | AY974070 |
|  | *Prevotella melaninogenica et rel.* | bacterium adhufec235 | AF132249 |
|  |  | *Prevotella intermedia* | AF414821 |
|  |  | *Prevotella albensis* | AJ011683 |
|  |  | *Prevotella melaninogenica* | L16469 |
|  |  | *Prevotella veroralis* | L16473 |
|  |  | *Prevotella disiens* | L16483 |
|  |  | uncultured bacterium B176 | AY916316 |
|  |  | uncultured bacterium M107 | AY916148 |
|  |  | Uncultured bacterium clone Eldhufec008 | AY919883 |
|  |  | Uncultured bacterium clone Eldhufec007 | AY919882 |
|  |  | Uncultured bacterium clone Eldhufec033 | AY919908 |
|  |  | Uncultured bacterium clone Eldhufec038 | AY919913 |
|  |  | Uncultured bacterium clone Eldhufec037 | AY919912 |
|  |  | Uncultured bacterium clone Eldhufec036 | AY919911 |
|  |  | Uncultured bacterium clone Eldhufec035 | AY919910 |
|  |  | Uncultured bacterium clone Eldhufec034 | AY919909 |
|  |  | Uncultured bacterium clone Eldhufec005 | AY919880 |
|  |  | Uncultured bacterium clone Eldhufec009 | AY919884 |
|  |  | Uncultured bacterium clone Eldhufec024 | AY919899 |
|  |  | Uncultured bacterium clone Eldhufec019 | AY919894 |
|  |  | uncultured bacterium HuJJ84 | AY684413 |
|  |  | *Prevotella* sp. BI-42 | AJ581354 |
|  | *Prevotella oralis et rel.* | *Prevotella oralis* | L16480 |
|  |  | *Prevotella* sp. CB25 | AB064924 |
|  |  | uncultured bacterium HuCC28 | AJ315483 |
|  |  | Uncultured bacterium clone Eldhufec011 | AY919886 |
|  |  | Uncultured bacterium clone Eldhufec043 | AY919918 |
|  |  | Uncultured bacterium clone Eldhufec015 | AY919890 |
|  |  | Uncultured bacterium clone Eldhufec017 | AY919892 |
|  |  | Uncultured bacterium clone Eldhufec012 | AY919887 |
|  |  | uncultured bacterium HuJJ29 | AY684415 |
|  |  | uncultured bacterium Adhufec036rbh | AY471699 |
|  | *Prevotella ruminicola et rel.* | *Prevotella ruminicola* | AF218618 |
|  |  | *Prevotella brevis* | AJ011682 |
|  |  | Uncultured bacterium clone Eldhufec028 | AY919903 |
|  | *Prevotella tannerae et rel.* | uncultured bacterium OLDC-G2 | AB099769 |
|  |  | uncultured bacterium OLDC-D5 | AB099768 |
|  |  | uncultured bacterium ME28 | AY916231 |
|  |  | Uncultured bacterium clone Eldhufec018 | AY919893 |
|  |  | Uncultured bacterium clone Eldhufec014 | AY919889 |
|  |  | Uncultured bacterium clone Eldhufec003 | AY919878 |
|  |  | uncultured bacterium cadhufec40c10 | AF530373 |
|  | *Tannerella et rel.* | bacterium adhufec77.25 | AF153865 |
|  |  | uncultured bacterium D487 | AY916372 |
|  |  | uncultured bacterium D761 | AY916386 |
|  |  | uncultured bacterium M070 | AY916146 |
|  |  | uncultured bacterium NG45 | AY916172 |
|  |  | uncultured bacterium NI77 | AY916176 |
|  |  | uncultured bacterium NO37 | AY916249 |
|  |  | uncultured bacterium NO50 | AY916251 |
|  |  | Uncultured bacterium clone Eldhufec010 | AY919885 |
|  |  | Uncultured bacterium clone Eldhufec041 | AY919916 |
|  |  | Uncultured bacterium clone Eldhufec006 | AY919881 |
|  |  | Uncultured bacterium clone Eldhufec004 | AY919879 |
|  |  | Uncultured bacterium clone Eldhufec023 | AY919898 |
|  |  | uncultured bacterium Adhufec048rbh | AY471701 |
|  | Unclutured *Bacteroidetes* | *Bacteroides* sp. CB40 | AB064919 |
| *Asteroleplasma* | *Asteroleplasma et rel.* | Uncultured bacterium UC7-11 | AJ608228 |
| *Bacilli* | *Aerococcus* | *Aerococcus viridans* | M58797 |
|  | *Bacillus et rel.* | *Bacillus halodurans* | AB013373 |
|  |  | *Bacillus subtilis* | AB018484 |
|  |  | *Bacillus pumilus* | AB020208 |
|  |  | *Bacillus flexus* | AB021185 |
|  |  | *Bacillus cereus* | AF076031 |
|  |  | *Bacillus sphaericus* | AF169495 |
|  |  | *Brevibacillus brevis* | AF424048 |
|  |  | *Bacillus megaterium* | D16273 |
|  |  | *Bacillus circulans* | D78312 |
|  |  | *Bacillus coagulans* | D78313 |
|  |  | *Aneurinibacillus aneurinolyticus* | D78455 |
|  |  | *Paenibacillus lautus* | D78472 |
|  |  | *Bacillus badius* | X77790 |
|  |  | *Paenibacillus durus* | X77846 |
|  | *Enterococcus* | *Enterococcus faecalis* | AB012212 |
|  |  | *Enterococcus faecium* | AB012213 |
|  |  | *Enterococcus gallinarum* | AF039898 |
|  |  | *Enterococcus casseliflavus* | AF039899 |
|  |  | *Enterococcus durans* | AF061000 |
|  |  | *Enterococcus avium* | AF061008 |
|  |  | *Enterococcus hirae* | AF061011 |
|  |  | uncultured bacterium cadhufec093h7 | AF530310 |
|  |  | uncultured bacterium (human infant) D8E | AF253331 |
|  | *Gemella* | *Gemella morbillorum* | L14327 |
|  | *Granulicatella* | Uncultured bacterium clone Eldhufec198 | AY920073 |
|  | *Lactobacillus gasseri et rel.* | *Lactobacillus gasseri* | AF243142 |
|  |  | *Lactobacillus jensenii* | AF243159 |
|  |  | *Lactobacillus crispatus* | AF257096 |
|  |  | *Lactobacillus johnsonii* | AJ002515 |
|  |  | *Lactobacillus delbrueckii* | AY050173 |
|  |  | *Lactobacillus acidophilus* | M58802 |
|  |  | *Lactobacillus amylovorus* | M58805 |
|  |  | *Lactobacillus helveticus* | X61141 |
|  |  | uncultured *Lactobacillus* sp. LabF368 | AF335876 |
|  |  | uncultured *Lactobacillus* sp. LabF93 | AF335911 |
|  |  | *Lactobacillus ultunensis* | AY253660 |
|  |  | *Lactobacillus kalixensis* | AY253657 |
|  | *Lactobacillus plantarum et rel.* | *Pediococcus acidilactici* | AB018213 |
|  |  | *Lactobacillus brevis* | AB024299 |
|  |  | *Lactobacillus mucosae* | AF126738 |
|  |  | *Lactobacillus rhamnosus* | AF243146 |
|  |  | *Lactobacillus paracasei* | AF243147 |
|  |  | *Lactobacillus fermentum* | AF243149 |
|  |  | *Lactobacillus vaginalis* | AF243177 |
|  |  | *Lactobacillus plantarum* | AJ271852 |
|  |  | *Lactobacillus casei* | AJ272201 |
|  |  | *Lactobacillus pentosus* | D79211 |
|  |  | *Lactobacillus reuteri* | L23507 |
|  |  | *Lactobacillus buchneri* | M58811 |
|  |  | *Pediococcus pentosaceus* | M58834 |
|  |  | *Lactobacillus oris* | X61131 |
|  |  | uncultured *Lactobacillus* sp. LabS14 | AF335913 |
|  |  | *Lactobacillus antri* | AY253659 |
|  |  | *Lactobacillus gastricus* | AY253658 |
|  |  | *Lactobacillus parabuchneri* | AB205056 |
|  | *Lactobacillus sakei et rel.* | *Lactobacillus sakei* | M58829 |
|  | *Lactobacillus salivarius et rel.* | *Lactobacillus salivarius* | AF420311 |
|  |  | *Lactobacillus ruminis* | M58828 |
|  | *Lactococcus* | *Lactococcus lactis* | AJ271851 |
|  |  | *Lactococcus* sp. 451 | AY762109 |
|  | *Staphylococcus* | *Staphylococcus aureus* | AF015929 |
|  |  | *Staphylococcus epidermidis* | D83362 |
|  |  | *Staphylococcus saccharolyticus* | L37602 |
|  | *Streptococcus bovis et rel.* | *Streptococcus equinus* | AB002514 |
|  |  | *Streptococcus uberis* | AB023573 |
|  |  | *Streptococcus agalactiae* | AB023574 |
|  |  | *Streptococcus pyogenes* | AF076028 |
|  |  | *Streptococcus bovis* | AF104109 |
|  |  | *Streptococcus infantarius* | AF177729 |
|  |  | *Streptococcus lutetiensis* | AF429763 |
|  |  | *Streptococcus salivarius* | M58839 |
|  |  | *Streptococcus thermophilus* | X59028 |
|  |  | uncultured bacterium OLDA-B7 | AB099789 |
|  |  | *Streptococcus equi subsp. zooepidemicus* | AB104843 |
|  |  | *Streptococcus equisimilis* | AJ314611 |
|  | *Streptococcus intermedius et rel.* | *Streptococcus intermedius* | AF104671 |
|  |  | *Streptococcus constellatus* | AF104676 |
|  |  | *Streptococcus anginosus* | AF145240 |
|  |  | *Streptococcus parasanguinis* | X53652 |
|  |  | Uncultured bacterium clone Eldhufec195 | AY920070 |
|  | *Streptococcus mitis et rel.* | *Streptococcus sanguis* | AF003928 |
|  |  | *Streptococcus mitis* | AF003929 |
|  |  | *Streptococcus oralis* | AF003932 |
|  |  | *Streptococcus viridans* | AF076036 |
|  |  | *Streptococcus mutans* | AJ243965 |
|  |  | uncultured *Streptococcus* sp. NB5C1 | AB064839 |
|  |  | bacterium ucfecDB2 | ARB_B5C8DA |
|  | *Weissella et rel.* | *Weissella cibaria* | AJ295989 |
|  |  | *Leuconostoc mesenteroides* | M23035 |
|  |  | *Weissella confusa* | M23036 |
|  |  | uncultured *Leuconostoc* sp. LabF165 | AF335897 |
| *Clostridium* cluster I | *Clostridium* | *Eubacterium multiforme* | AB018184 |
|  |  | *Clostridium paraputrificum* | AB032556 |
|  |  | *Clostridium perfringens* | AB045282 |
|  |  | *Clostridium botulinum* | AF105402 |
|  |  | *Sarcina ventriculi* | AF110272 |
|  |  | *Clostridium putrefaciens* | AF127024 |
|  |  | *Clostridium subterminale* | AF241842 |
|  |  | *Clostridium butyricum* | AJ002592 |
|  |  | *Clostridium tertium* | AJ245413 |
|  |  | *Clostridium tyrobutyricum* | L08062 |
|  |  | *Eubacterium moniliforme* | L34622 |
|  |  | *Clostridium cadaveris* | M59086 |
|  |  | *Clostridium fallax* | M59088 |
|  |  | *Clostridium cochlearium* | M59093 |
|  |  | *Clostridium limosum* | M59096 |
|  |  | *Clostridium malenominatum* | M59099 |
|  |  | *Clostridium paraperfringens* | M59102 |
|  |  | *Clostridium sporogenes* | M59115 |
|  |  | *Clostridium acetobutylicum* | S46735 |
|  |  | *Clostridium septicum* | U59278 |
|  |  | *Clostridium barati* | X68174 |
|  |  | *Clostridium beijerinckii* | X68179 |
|  |  | *Clostridium celatum* | X77844 |
|  |  | *Clostridium sartagoformum* | Y18175 |
|  |  | Uncultured bacterium clone Eldhufec341 | AY920216 |
|  |  | *Eubacterium budayi* | AB018183 |
|  |  | *Eubacterium nitritogenes* | AB018185 |
| *Clostridium* cluster III | *Clostridium stercorarium et rel.* | uncultured bacterium B839 | AY916322 |
|  |  | uncultured bacterium D145 | AY916358 |
|  |  | uncultured bacterium LE17 | AY916205 |
|  |  | Uncultured bacterium clone Eldhufec339 | AY920214 |
|  |  | Uncultured bacterium UC7-82 | AJ608246 |
|  | *Clostridium thermocellum et rel.* | uncultured bacterium C288 | AY916331 |
|  |  | Uncultured bacterium clone Eldhufec338 | AY920213 |
| *Clostridium* cluster IV | *Anaerotruncus colihominis et rel.* | bacterium adhufec101 | AF132235 |
|  |  | uncultured Gram-positive bacterium NO2-2 | AB064805 |
|  |  | uncultured bacterium D577 | AY916375 |
|  |  | uncultured bacterium LF02 | AY916207 |
|  |  | uncultured bacterium LL29 | AY916260 |
|  |  | uncultured bacterium LL87 | AY916261 |
|  |  | uncultured bacterium HuCA1 | AJ408957 |
|  |  | Uncultured bacterium clone Eldhufec246 | AY920121 |
|  |  | Uncultured bacterium clone Eldhufec211 | AY920086 |
|  |  | Uncultured bacterium clone Eldhufec214 | AY920089 |
|  |  | Uncultured bacterium clone Eldhufec215 | AY920090 |
|  |  | Uncultured bacterium clone Eldhufec265 | AY920140 |
|  |  | Uncultured bacterium clone Eldhufec270 | AY920145 |
|  |  | *Anaerotruncus colihominis* | AJ315980 |
|  | *Clostridium cellulosi rel.* | uncultured human gut bacterium JW1B12 | AB080849 |
|  |  | uncultured bacterium OLDB-E4 | AB099734 |
|  |  | uncultured bacterium C342 | AY916333 |
|  |  | uncultured bacterium D036 | AY916351 |
|  |  | uncultured bacterium K507 | AY916200 |
|  |  | uncultured bacterium LZ45 | AY916188 |
|  |  | uncultured bacterium M490 | AY916159 |
|  |  | uncultured bacterium M511 | AY916162 |
|  |  | uncultured bacterium MH24 | AY916292 |
|  |  | uncultured bacterium Z456 | AY916179 |
|  |  | uncultured bacterium D626 | AY916378 |
|  |  | Uncultured bacterium clone Eldhufec236 | AY920111 |
|  |  | Uncultured bacterium clone Eldhufec212 | AY920087 |
|  |  | Uncultured bacterium clone Eldhufec213 | AY920088 |
|  |  | Uncultured bacterium clone Eldhufec273 | AY920148 |
|  |  | Uncultured bacterium clone Eldhufec249 | AY920124 |
|  |  | Uncultured bacterium UC7-44 | AJ608241 |
|  |  | Uncultured bacterium UC7-69 | AJ608244 |
|  |  | uncultured bacterium cadhufec022h7 | AF530299 |
|  |  | uncultured bacterium ABLCf36 | AF499903 |
|  |  | uncultured bacterium HuAC35 | AY684394 |
|  |  | uncultured bacterium Adhufec106abh | AY471691 |
|  | *Clostridium leptum et rel.* | *Clostridium leptum* | M59095 |
|  |  | *Clostridium sporosphaeroides* | M59116 |
|  |  | uncultured human gut bacterium JW1C7 | AB080848 |
|  |  | uncultured bacterium C464 | AY916336 |
|  |  | uncultured bacterium C735 | AY916345 |
|  |  | uncultured bacterium K288 | AY916193 |
|  |  | uncultured bacterium HuCA24 | AJ408976 |
|  |  | Uncultured bacterium clone Eldhufec221 | AY920096 |
|  |  | Uncultured bacterium UC7-14 | AJ608230 |
|  |  | uncultured bacterium adhufec168 | AF132242 |
|  |  | *Ruminococcus* sp. 16442 | AJ318889 |
|  | *Clostridium orbiscindens et rel.* | *Clostridium orbiscindens* | Y18187 |
|  |  | human intestinal firmicute CJ36 | AB080896 |
|  |  | human intestinal firmicute CJ31 | AB080897 |
|  |  | uncultured human gut bacterium JW1D6 | AB080858 |
|  |  | uncultured human gut bacterium JW2G1 | AB080857 |
|  |  | uncultured human gut bacterium JW1G9 | AB080856 |
|  |  | uncultured human gut bacterium JW2A8 | AB080855 |
|  |  | uncultured bacterium OLDA-F4 | AB099727 |
|  |  | uncultured bacterium B632 | AY916320 |
|  |  | uncultured bacterium D330 | AY916365 |
|  |  | uncultured bacterium D465 | AY916371 |
|  |  | uncultured bacterium D588 | AY916376 |
|  |  | uncultured bacterium G267 | AY916285 |
|  |  | uncultured bacterium K351 | AY916196 |
|  |  | uncultured bacterium LV67 | AY916184 |
|  |  | uncultured bacterium M510 | AY916161 |
|  |  | uncultured bacterium W074 | AY916213 |
|  |  | uncultured bacterium HuCB24 | AJ408998 |
|  |  | Uncultured bacterium clone Eldhufec218 | AY920093 |
|  |  | Uncultured bacterium clone Eldhufec272 | AY920147 |
|  |  | Uncultured bacterium clone Eldhufec262 | AY920137 |
|  |  | Uncultured bacterium clone Eldhufec264 | AY920139 |
|  |  | Uncultured bacterium clone Eldhufec267 | AY920142 |
|  |  | Uncultured bacterium clone Eldhufec229 | AY920104 |
|  |  | uncultured bacterium cadhufec074h7 | AF530307 |
|  |  | *Bacteroides capillosus* | AY136666 |
|  |  | uncultured bacterium Adhufec102rbh | AY471712 |
|  | *Eubacterium siraeum et rel.* | *Eubacterium siraeum* | L34625 |
|  |  | uncultured bacterium B025 | AY916313 |
|  |  | Uncultured bacterium clone Eldhufec237 | AY920112 |
|  |  | Uncultured bacterium clone Eldhufec239 | AY920114 |
|  |  | Uncultured bacterium UC7-117 | AJ608247 |
|  |  | uncultured bacterium Adhufec058abh | AY471683 |
|  | *Faecalibacterium prausnitzii et rel.* | bacterium adhufec113 | AF132236 |
|  |  | butyrate-producing bacterium A2-165 | AJ270469 |
|  |  | butyrate-producing bacterium L2-6 | AJ270470 |
|  |  | *Faecalibacterium prausnitzii* | AJ413954 |
|  |  | uncultured bacterium KM82 | AY916180 |
|  |  | uncultured bacterium KP66 | AY916136 |
|  |  | uncultured bacterium HuCA25 | AJ408973 |
|  |  | uncultured bacterium HuCA11 | AJ408966 |
|  |  | Uncultured bacterium clone Eldhufec238 | AY920113 |
|  |  | Uncultured bacterium clone Eldhufec226 | AY920101 |
|  |  | Uncultured bacterium clone Eldhufec227 | AY920102 |
|  |  | Uncultured bacterium clone Eldhufec288 | AY920163 |
|  |  | Uncultured bacterium clone Eldhufec228 | AY920103 |
|  |  | Uncultured bacterium clone Eldhufec259 | AY920134 |
|  |  | Uncultured bacterium clone Eldhufec261 | AY920136 |
|  |  | Uncultured bacterium clone Eldhufec276 | AY920151 |
|  |  | Uncultured bacterium clone Eldhufec282 | AY920157 |
|  |  | Uncultured bacterium clone Eldhufec256 | AY920131 |
|  |  | Uncultured bacterium clone Eldhufec255 | AY920130 |
|  |  | Uncultured bacterium clone Eldhufec252 | AY920127 |
|  |  | Uncultured bacterium clone Eldhufec281 | AY920156 |
|  |  | Uncultured bacterium clone Eldhufec251 | AY920126 |
|  |  | uncultured bacterium adhufec08.25 | AF153871 |
|  |  | uncultured bacterium A10 | AF052411 |
|  |  | uncultured bacterium Adhufec010abh | AY471671 |
|  |  | uncultured bacterium Adhufec055abh | AY471682 |
|  |  | uncultured bacterium Adhufec052abh | AY471681 |
|  |  | uncultured bacterium Adhufec064rbh | AY471704 |
|  |  | uncultured bacterium Adhufec057rbh | AY471702 |
|  |  | uncultured bacterium Adhufec107rbh | AY471714 |
|  | *Oscillospira guillermondii et rel.* | bacterium adhufec269 | AF132255 |
|  |  | uncultured human gut bacterium JW1C11 | AB080854 |
|  |  | uncultured bacterium OLDA-D11 | AB099726 |
|  |  | uncultured bacterium OLDC-D12 | AB099725 |
|  |  | uncultured bacterium OLDA-H2 | AB099721 |
|  |  | uncultured bacterium A051 | AY916256 |
|  |  | uncultured bacterium B811 | AY916321 |
|  |  | uncultured bacterium C574 | AY916337 |
|  |  | uncultured bacterium D134 | AY916357 |
|  |  | uncultured bacterium D288 | AY916364 |
|  |  | uncultured bacterium D440 | AY916370 |
|  |  | uncultured bacterium LE02 | AY916204 |
|  |  | uncultured bacterium MA30 | AY916224 |
|  |  | uncultured bacterium MM71 | AY916303 |
|  |  | uncultured bacterium V239 | AY916276 |
|  |  | uncultured bacterium HuCB7 | AJ408991 |
|  |  | Uncultured bacterium clone Eldhufec241 | AY920116 |
|  |  | Uncultured bacterium clone Eldhufec223 | AY920098 |
|  |  | Uncultured bacterium clone Eldhufec257 | AY920132 |
|  |  | Uncultured bacterium clone Eldhufec301 | AY920176 |
|  |  | Uncultured bacterium clone Eldhufec285 | AY920160 |
|  |  | Uncultured bacterium clone Eldhufec283 | AY920158 |
|  |  | uncultured bacterium cadhufec121h7 | AF530315 |
|  |  | uncultured bacterium Adhufec002abh | AY471669 |
|  |  | uncultured bacterium Adhufec044abh | AY471679 |
|  | Outgrouping *Clostridium* cluster IV | uncultured bacterium C747 | AY916347 |
|  |  | uncultured bacterium LD25 | AY916202 |
|  |  | uncultured bacterium V366 | AY916279 |
|  |  | Uncultured bacterium clone Eldhufec318 | AY920193 |
|  |  | Uncultured bacterium clone Eldhufec320 | AY920195 |
|  |  | Uncultured bacterium clone Eldhufec321 | AY920196 |
|  |  | Uncultured bacterium clone Eldhufec319 | AY920194 |
|  | *Papillibacter cinnamivorans et rel.* | bacterium adhufec296 | AF132258 |
|  |  | butyrate-producing bacterium A2-207 | AJ270471 |
|  |  | uncultured Gram-positive bacterium NB5F9 | AB064783 |
|  |  | uncultured bacterium ZO15 | AY916177 |
|  |  | Uncultured bacterium clone Eldhufec233 | AY920108 |
|  |  | Uncultured bacterium clone Eldhufec245 | AY920120 |
|  |  | Uncultured bacterium clone Eldhufec258 | AY920133 |
|  |  | uncultured bacterium cadhufec32c10 | AF530372 |
|  | *Ruminococcus bromii et rel.* | *Ruminococcus bromii* | L76600 |
|  |  | uncultured bacterium HuCB2 | AJ408987 |
|  |  | Uncultured bacterium clone Eldhufec230 | AY920105 |
|  |  | Uncultured bacterium clone Eldhufec291 | AY920166 |
|  |  | Uncultured bacterium clone Eldhufec225 | AY920100 |
|  |  | Uncultured bacterium clone Eldhufec291 | AY920166 |
|  |  | uncultured bacterium cadhufec021h7 | AF530298 |
|  |  | uncultured bacterium Adhufec014rbh | AY471694 |
|  | *Ruminococcus callidus et rel.* | *Ruminococcus flavefaciens* | AF030446 |
|  |  | *Ruminococcus albus* | AF030451 |
|  |  | *Ruminococcus callidus* | L76596 |
|  |  | *Clostridium methylpentosum* | Y18181 |
|  |  | uncultured Gram-positive bacterium NS4G9 | AB064811 |
|  |  | uncultured *Ruminococcus* sp. NO11 | AB064808 |
|  |  | uncultured bacterium D005 | AY916350 |
|  |  | uncultured bacterium D739 | AY916385 |
|  |  | uncultured bacterium D789 | AY916389 |
|  |  | uncultured bacterium MF20 | AY916235 |
|  |  | uncultured bacterium MH26 | AY916293 |
|  |  | Uncultured bacterium clone Eldhufec235 | AY920110 |
|  |  | Uncultured bacterium clone Eldhufec284 | AY920159 |
|  |  | Uncultured bacterium clone Eldhufec250 | AY920125 |
|  | *Sporobacter termitidis rel.* | bacterium adhufec311 | AF132261 |
|  |  | bacterium adhufec108 | AF132283 |
|  |  | uncultured bacterium OLDC-E8 | AB099728 |
|  |  | uncultured bacterium C352 | AY916334 |
|  |  | uncultured bacterium C354 | AY916335 |
|  |  | uncultured bacterium C727 | AY916344 |
|  |  | uncultured bacterium D762 | AY916387 |
|  |  | uncultured bacterium L495 | AY916281 |
|  |  | uncultured bacterium LO41 | AY916265 |
|  |  | uncultured bacterium LQ71 | AY916268 |
|  |  | uncultured bacterium LY18 | AY916187 |
|  |  | Uncultured bacterium clone Eldhufec210 | AY920085 |
|  |  | Uncultured bacterium clone Eldhufec290 | AY920165 |
|  |  | Uncultured bacterium clone Eldhufec274 | AY920149 |
|  |  | Uncultured bacterium clone Eldhufec231 | AY920106 |
|  |  | Uncultured bacterium clone Eldhufec294 | AY920169 |
|  |  | Uncultured bacterium clone Eldhufec216 | AY920091 |
|  |  | Uncultured bacterium clone Eldhufec217 | AY920092 |
|  |  | Uncultured bacterium clone Eldhufec287 | AY920162 |
|  |  | Uncultured bacterium clone Eldhufec220 | AY920095 |
|  |  | Uncultured bacterium clone Eldhufec232 | AY920107 |
|  |  | Uncultured bacterium UC7-1 | AJ608220 |
|  | *Subdoligranulum variable at rel.* | bacterium adhufec13 | AF132237 |
|  |  | uncultured Gram-positive bacterium NO2- | AB064804 |
|  |  | uncultured Gram-positive bacterium NB5C6 | AB064803 |
|  |  | human intestinal firmicute CJ7 | AB080895 |
|  |  | uncultured human gut bacterium JW1D4 | AB080847 |
|  |  | uncultured bacterium LC79 | AY916201 |
|  |  | uncultured bacterium M479 | AY916158 |
|  |  | uncultured bacterium HuCB5 | AJ408989 |
|  |  | Uncultured bacterium clone Eldhufec243 | AY920118 |
|  |  | Uncultured bacterium clone Eldhufec222 | AY920097 |
|  |  | Uncultured bacterium clone Eldhufec224 | AY920099 |
|  |  | Uncultured bacterium clone Eldhufec260 | AY920135 |
|  |  | Uncultured bacterium clone Eldhufec302 | AY920177 |
|  |  | Uncultured bacterium clone Eldhufec268 | AY920143 |
|  |  | uncultured bacterium cadhufec068h7 | AF530306 |
|  |  | uncultured bacterium cadhufec066h7 | AF530305 |
|  |  | uncultured bacterium ABLCf22 | AF499901 |
|  |  | *Subdoligranulum variabile* | AJ518869 |
| *Clostridium* cluster IX | *Dialister* | *Dialister pneumosintes* | X82500 |
|  |  | uncultured Gram-positive bacterium NS2B1 | AB064859 |
|  |  | Uncultured bacterium clone Eldhufec091 | AY919966 |
|  |  | Uncultured bacterium clone Eldhufec093 | AY919968 |
|  |  | Uncultured bacterium clone Eldhufec089 | AY919964 |
|  |  | Uncultured bacterium clone Eldhufec096 | AY919971 |
|  |  | uncultured bacterium B856 | AY984881 |
|  |  | uncultured bacterium MG10 | AY982155 |
|  | *Megamonas hypermegale et rel.* | *Megamonas hypermegale* | AJ420107 |
|  |  | human intestinal firmicute CB15 | AB064931 |
|  |  | uncultured bacterium cadhufec43c10 | AF530374 |
|  | *Megasphaera elsdenii et rel.* | *Megasphaera elsdenii* | AF283705 |
|  |  | uncultured bacterium OLDC-D10 | AB099774 |
|  |  | uncultured bacterium HuCB85 | AJ409007 |
|  |  | Uncultured bacterium clone Eldhufec098 | AY919973 |
|  |  | uncultured bacterium inhufecA-11 | AY328359 |
|  | *Mitsuokella multiacida et rel.* | *Selenomonas ruminantium* | AB017195 |
|  |  | *Mitsuokella multiacida* | X81878 |
|  |  | uncultured Gram-positive bacterium NB5E1 | AB064853 |
|  |  | uncultured bacterium OLDC-C6 | AB099772 |
|  | *Peptococcus niger et rel.* | *Peptococcus niger* | X55797 |
|  |  | uncultured bacterium D393 | AY916367 |
|  |  | uncultured bacterium MH31 | AY916294 |
|  |  | uncultured bacterium V247 | AY916277 |
|  |  | Uncultured bacterium clone Eldhufec095 | AY919970 |
|  |  | uncultured bacterium HuDI10 | AY862394 |
|  | *Phascolarctobacterium faecium et rel.* | bacterium adhufec395 | AF132234 |
|  |  | *Acidaminococcus fermentans* | X65935 |
|  |  | uncultured Gram-positive bacterium NB4G9 | AB064849 |
|  |  | uncultured bacterium OLDB-D6 | AB099771 |
|  |  | uncultured bacterium OLDB-B2 | AB099753 |
|  |  | uncultured bacterium D115 | AY916356 |
|  |  | Uncultured bacterium clone Eldhufec097 | AY919972 |
|  |  | Uncultured bacterium clone Eldhufec094 | AY919969 |
|  |  | uncultured bacterium cadhufec137c10 | AF530370 |
|  | Uncultured Selenomonadaceae | uncultured bacterium HuAC20 | AY684401 |
|  | *Veillonella* | *Veillonella dispar* | AF439639 |
|  |  | *Veillonella parvula* | AF439640 |
|  |  | *Veillonella atypica* | AF439641 |
|  |  | uncultured bacterium ABLCf8 | AF499900 |
| *Clostridium* cluster XI | *Anaerovorax odorimutans rel.* | uncultured Gram-positive bacterium NO2-6 | AB064863 |
|  |  | uncultured human gut bacterium JW1G2 | AB080883 |
|  |  | uncultured bacterium LN56 | AY916263 |
|  |  | uncultured bacterium MO17 | AY916142 |
|  |  | uncultured bacterium MH36 | AY916295 |
|  |  | uncultured bacterium P615 | AY916312 |
|  |  | Uncultured bacterium clone Eldhufec185 | AY920060 |
|  |  | Uncultured bacterium clone Eldhufec187 | AY920062 |
|  |  | Uncultured bacterium clone Eldhufec186 | AY920061 |
|  |  | uncultured bacterium HuJJ43 | AY684403 |
|  |  | uncultured bacterium HuRC86 | AY684402 |
|  | *Clostridium difficile et rel.* | *Clostridium hiranonis* | AB023970 |
|  |  | *Clostridium difficile* | AF072473 |
|  |  | *Clostridium bifermentans* | AF320283 |
|  |  | *Clostridium glycolicum* | AY007244 |
|  |  | *Clostridium sticklandii* | L04167 |
|  |  | *Clostridium sordellii* | M59105 |
|  |  | *Eubacterium tenue* | M59118 |
|  |  | *Clostridium irregularis* | X73447 |
|  |  | *Clostridium ghoni* | X73451 |
|  |  | uncultured Gram-positive bacterium NS1E9 | AB064876 |
|  |  | uncultured *Clostridium* sp. NB4D7 | AB064872 |
|  |  | uncultured bacterium OLDB-G12 | AB099796 |
|  |  | uncultured bacterium M364 | AY916153 |
|  |  | Uncultured bacterium clone Eldhufec189 | AY920064 |
|  |  | uncultured bacterium LCLC73 | AF499844 |
|  |  | uncultured bacterium LCLC21 | AF499843 |
|  |  | *Clostridium bartlettii* | AY438672 |
|  | *Clostridium felsineum* | *Clostridium felsineum* | X77851 |
|  | *Peptostreptococcus anaerobius et rel.* | *Peptostreptococcus anaerobius* | D14150 |
|  |  | uncultured bacterium C120 | AY916327 |
| *Clostridium* cluster XIII | *Peptostreptococcus micros et rel.* | *Peptoniphilus asaccharolyticus* | D14138 |
|  |  | *Anaerococcus prevotii* | D14139 |
|  |  | *Anaerococcus hydrogenalis* | D14140 |
|  |  | *Peptostreptococcus micros* | D14143 |
|  |  | *Peptoniphilus indolicus* | D14147 |
|  |  | *Finegoldia magna* | D14149 |
|  |  | uncultured bacterium G170 | AY981208 |
|  | *Tissierella* | *Tissierella praeacuta* | X80833 |
| *Clostridium* cluster XIVa | *Acetitomaculum ruminis rel.* | bacterium adhufec250 | AF132253 |
|  |  | uncultured bacterium D416 | AY916368 |
|  |  | uncultured bacterium LP40 | AY916266 |
|  |  | uncultured bacterium M977 | AY916221 |
|  |  | Uncultured bacterium clone Eldhufec157 | AY920032 |
|  |  | Uncultured bacterium clone Eldhufec120 | AY919995 |
|  |  | Uncultured bacterium clone Eldhufec117 | AY919992 |
|  |  | Uncultured bacterium clone Eldhufec110 | AY919985 |
|  |  | Uncultured bacterium clone Eldhufec103 | AY919978 |
|  |  | uncultured bacterium HuDI84 | AY684365 |
|  | *Anaerostipes caccae et rel.* | *Clostridium indolis* | AF028351 |
|  |  | bacterium adhufec25 | AF132254 |
|  |  | *Anaerostipes caccae* | AJ270487 |
|  |  | uncultured Gram-positive bacterium NB2G8 | AB064714 |
|  |  | uncultured Gram-positive bacterium NO2-5 | AB064713 |
|  |  | uncultured human gut bacterium JW2C7 | AB080875 |
|  |  | uncultured bacterium HuCA20 | AJ408972 |
|  | *Bryantella formatexigens et rel.* | bacterium adhufec40 | AF132270 |
|  |  | *Eubacterium cellulosolvens* | L34613 |
|  |  | uncultured Gram-positive bacterium NS2F9 | AB064773 |
|  |  | *Ruminococcus* sp. CO28 | AB064891 |
|  |  | uncultured bacterium M629 | AY916166 |
|  |  | uncultured bacterium M963 | AY916220 |
|  |  | uncultured bacterium ME57 | AY916233 |
|  |  | uncultured bacterium MF29 | AY916238 |
|  |  | uncultured bacterium P315 | AY916311 |
|  |  | Uncultured bacterium clone Eldhufec135 | AY920010 |
|  |  | Uncultured bacterium clone Eldhufec152 | AY920027 |
|  |  | Uncultured bacterium UC7-3 | AJ608221 |
|  |  | Uncultured bacterium UC7-50 | AJ608242 |
|  |  | uncultured bacterium cadhufec56c10 | AF530376 |
|  |  | uncultured bacterium ABLCf44 | AF499907 |
|  |  | *Bryantella formatexigens* | AJ318527 |
|  |  | uncultured bacterium HuRC75 | AY684376 |
|  |  | uncultured bacterium Adhufec124abh | AY471692 |
|  | *Butyrivibrio crossotus et rel.* | bacterium adhufec406 | AF132269 |
|  |  | *Eubacterium ramulus* | AJ011522 |
|  |  | *Butyrivibrio crossotus* | X89981 |
|  |  | uncultured bacterium D680 | AY916379 |
|  |  | uncultured bacterium D692 | AY916380 |
|  |  | uncultured bacterium D726 | AY916383 |
|  |  | uncultured bacterium D738 | AY916384 |
|  |  | uncultured bacterium MG71 | AY916289 |
|  |  | Uncultured bacterium clone Eldhufec138 | AY920013 |
|  |  | Uncultured bacterium clone Eldhufec155 | AY920030 |
|  |  | Uncultured bacterium clone Eldhufec116 | AY919991 |
|  |  | Uncultured bacterium clone Eldhufec114 | AY919989 |
|  |  | Uncultured bacterium clone Eldhufec112 | AY919987 |
|  |  | Uncultured bacterium clone Eldhufec147 | AY920022 |
|  |  | Uncultured bacterium clone Eldhufec244 | AY920119 |
|  |  | uncultured bacterium Adhufec023abh | AY471673 |
|  |  | uncultured bacterium Adhufec112rbh | AY471715 |
|  |  | uncultured bacterium Muc3-1 | AY451999 |
|  | *Clostridium glycyrrhizinilyticum et rel.* | uncultured human gut bacterium JW1G3 | AB080863 |
|  |  | uncultured human gut bacterium JW1A12 | AB080860 |
|  |  | uncultured bacterium NP09 | AY916252 |
|  |  | uncultured bacterium HuCC43 | AJ315487 |
|  |  | Uncultured bacterium clone Eldhufec125 | AY920000 |
|  |  | Uncultured bacterium clone Eldhufec123 | AY919998 |
|  |  | uncultured bacterium cadhufec69c10 | AF530380 |
|  |  | uncultured bacterium cadhufec101h7 | AF530314 |
|  |  | uncultured bacterium HuRC12 | AY684370 |
|  |  | *Clostridium glycyrrhizinilyticum* | AB233029 |
|  | *Clostridium lactifermentans et rel.* | uncultured bacterium G075 | AY916283 |
|  |  | uncultured bacterium K305 | AY916194 |
|  |  | uncultured bacterium NK21 | AY916240 |
|  |  | Uncultured bacterium clone Eldhufec141 | AY920016 |
|  |  | Uncultured bacterium clone Eldhufec182 | AY920057 |
|  |  | Uncultured bacterium clone Eldhufec183 | AY920058 |
|  |  | uncultured bacterium HuDI72 | AY684405 |
|  |  | uncultured bacterium HuDI23 | AY684406 |
|  |  | *Clostridium lactatifermentans* | AY033434 |
|  | *Clostridium nexile et rel.* | butyrate-producing bacterium A2-231 | AJ270484 |
|  |  | *Clostridium nexile* | X73443 |
|  |  | uncultured Gram-positive bacterium NB4C3 | AB064747 |
|  |  | uncultured Gram-positive bacterium NO2-4 | AB064746 |
|  |  | uncultured Gram-positive bacterium NO31 | AB064743 |
|  |  | uncultured Gram-positive bacterium NO81 | AB064742 |
|  |  | uncultured bacterium OLDB-F3 | AB099735 |
|  |  | uncultured bacterium cadhufec20a04 | AF530331 |
|  |  | uncultured bacterium LCRC24 | AF499855 |
|  |  | uncultured bacterium ABLC1 | AF499881 |
|  |  | uncultured bacterium ABLCf89 | AF499909 |
|  | *Clostridium sphenoides et rel.* | bacterium A21 | AF052418 |
|  |  | bacterium A54 | AF052421 |
|  |  | bacterium adhufec382 | AF132267 |
|  |  | *Clostridium sphenoides* | X73449 |
|  |  | uncultured Gram-positive bacterium NB2A8 | AB064730 |
|  |  | uncultured Gram-positive bacterium NO2-2 | AB064727 |
|  |  | uncultured bacterium HuCA27 | AJ408978 |
|  |  | uncultured bacterium HuCA19 | AJ408971 |
|  |  | uncultured bacterium HuCA17 | AJ408969 |
|  |  | uncultured bacterium LCLC63 | AF499839 |
|  |  | uncultured bacterium LCLC23 | AF499838 |
|  |  | uncultured bacterium ABLC30 | AF499880 |
|  |  | uncultured bacterium ABLCf11 | AF499906 |
|  |  | *Clostridium hathewayi* | AJ311620 |
|  |  | uncultured bacterium Adhufec088khh | AY471662 |
|  | *Clostridium symbiosum et rel.* | *Clostridium clostridiiformes* | M59089 |
|  |  | *Clostridium symbiosum* | M59112 |
|  |  | *Clostridium* sp. CJ23 | AB080893 |
|  |  | uncultured bacterium B147 | AY916315 |
|  |  | uncultured bacterium B395 | AY916317 |
|  |  | uncultured bacterium B840 | AY916323 |
|  |  | uncultured bacterium K375 | AY916197 |
|  |  | uncultured bacterium L812 | AY916282 |
|  |  | uncultured bacterium MB66 | AY916225 |
|  |  | uncultured bacterium MD61 | AY916228 |
|  |  | uncultured bacterium MI29 | AY916299 |
|  |  | uncultured bacterium HuCC34 | AJ315486 |
|  |  | Uncultured bacterium clone Eldhufec149 | AY920024 |
|  |  | Uncultured bacterium clone Eldhufec115 | AY919990 |
|  |  | Uncultured bacterium clone Eldhufec100 | AY919975 |
|  |  | uncultured bacterium inhufecA-32 | AY328366 |
|  |  | uncultured bacterium LCTI22 | AF499870 |
|  |  | *Clostridium asparagiforme* | AJ582080 |
|  |  | *Clostridium bolteae* | AJ508452 |
|  |  | butyrate-producing bacterium M62/1 | AY305309 |
|  |  | uncultured bacterium M985 | AY983861 |
|  | *Coprococcus catus et rel.* | butyrate-producing bacterium L2-10 | AJ270486 |
|  |  | uncultured human gut bacterium JW1B8 | AB080861 |
|  |  | uncultured bacterium KO89 | AY916135 |
|  |  | uncultured bacterium NW71 | AY916309 |
|  |  | Uncultured bacterium UC7-62 | AJ608243 |
|  |  | uncultured bacterium cadhufec098h7 | AF530312 |
|  |  | *Coprococcus catus* | AB038359 |
|  | *Coprococcus eutactus et rel.* | *Eubacterium ruminantium* | AB008552 |
|  |  | bacterium A57 | AF052422 |
|  |  | bacterium adhufec157 | AF132241 |
|  |  | butyrate-producing bacterium A2-166 | AJ270489 |
|  |  | *Coprococcus eutactus* | D14148 |
|  |  | uncultured Ruminococcus sp. NB2B8 | AB064761 |
|  |  | Uncultured bacterium UC7-8 | AJ608226 |
|  | *Dorea formicigenerans et rel.* | *Clostridium scindens* | AB020727 |
|  |  | *Clostridium hylemonae* | AB023972 |
|  |  | bacterium A71 | AF052423 |
|  |  | *Dorea formicigenerans* | L34619 |
|  |  | uncultured Gram-positive bacterium NS2C1 | AB064738 |
|  |  | human intestinal firmicute CO39 | AB064889 |
|  |  | uncultured human gut bacterium JW1H4b | AB080873 |
|  |  | uncultured bacterium KW79 | AY916215 |
|  |  | uncultured bacterium N874 | AY916190 |
|  |  | uncultured bacterium HuCB21 | AJ408996 |
|  |  | *Dorea longicatena* | AJ132842 |
|  | *Eubacterium hallii et rel.* | *Eubacterium hallii* | L34621 |
|  |  | uncultured bacterium HuCB26 | AJ409000 |
|  |  | uncultured bacterium HuCC15 | AJ315482 |
|  |  | uncultured bacterium Adhufec106khh | AY471665 |
|  |  | uncultured bacterium Adhufec127rbh | AY471720 |
|  |  | bacterium ucfecDC6 |  |
|  | *Eubacterium rectale et rel.* | *Butyrivibrio fibrisolvens* | AB004910 |
|  |  | *Eubacterium rectale* | L34627 |
|  |  | uncultured bacterium D522 | AY916373 |
|  |  | uncultured bacterium M372 | AY916154 |
|  |  | uncultured bacterium HuCB37 | AJ409004 |
|  |  | uncultured bacterium HuCA8 | AJ408964 |
|  |  | Uncultured bacterium clone Eldhufec130 | AY920005 |
|  |  | Uncultured bacterium clone Eldhufec121 | AY919996 |
|  |  | *Lachnobacterium* sp. wal 14165 | AJ518873 |
|  |  | uncultured bacterium A22 | AF052419 |
|  | *Eubacterium ventriosum et rel.* | bacterium adhufec335 | AF132262 |
|  |  | *Eubacterium ventriosum* | L34421 |
|  |  | uncultured bacterium D177 | AY916360 |
|  | *Lachnobacillus bovis et rel.* | bacterium A11 | AF052412 |
|  |  | bacterium adhufec68 | AF132278 |
|  |  | uncultured bacterium B558 | AY916318 |
|  |  | uncultured bacterium D695 | AY916382 |
|  |  | uncultured bacterium ME11 | AY916230 |
|  |  | Uncultured bacterium clone Eldhufec139 | AY920014 |
|  |  | Uncultured bacterium clone Eldhufec137 | AY920012 |
|  |  | Uncultured bacterium clone Eldhufec153 | AY920028 |
|  |  | Uncultured bacterium clone Eldhufec118 | AY919993 |
|  | *Lachnospira pectinoschiza et rel.* | *Lachnospira pectinoschiza* | L14675 |
|  |  | *Eubacterium eligens* | L34420 |
|  |  | uncultured bacterium LZ58 | AY916189 |
|  |  | Uncultured bacterium clone Eldhufec140 | AY920015 |
|  |  | Uncultured bacterium clone Eldhufec105 | AY919980 |
|  |  | Uncultured bacterium UC7-131 | AJ608250 |
|  |  | uncultured bacterium ABLCf6 | AF499905 |
|  | Outgrouping *Clostridium*  cluster XIVa | bacterium adhufec236 | AF132250 |
|  |  | bacterium adhufec295 | AF132257 |
|  |  | bacterium adhufec405 | AF132268 |
|  |  | bacterium adhufec52 | AF132274 |
|  |  | *Clostridium aminovalericum* | M23929 |
|  |  | uncultured human gut bacterium JW1C1 | AB080872 |
|  |  | uncultured human gut bacterium JW1D8 | AB080871 |
|  |  | uncultured bacterium LL95 | AY916262 |
|  |  | uncultured bacterium MK42 | AY916301 |
|  |  | uncultured bacterium N322 | AY916273 |
|  |  | uncultured bacterium NL43 | AY916244 |
|  |  | uncultured bacterium V213 | AY916275 |
|  |  | uncultured bacterium HuCB56 | AJ409006 |
|  |  | Uncultured bacterium clone Eldhufec129 | AY920004 |
|  |  | Uncultured bacterium clone Eldhufec184 | AY920059 |
|  |  | Uncultured bacterium clone Eldhufec111 | AY919986 |
|  |  | butyrate-producing bacterium SS3/4 | AY305316 |
|  |  | uncultured bacterium HuAC36 | AY684386 |
|  |  | uncultured bacterium Adhufec004abh | AY471670 |
|  |  | uncultured bacterium Adhufec071rbh | AY471707 |
|  |  | uncultured bacterium Muc3-13 | AY452004 |
|  | *Roseburia intestinalis et rel.* | butyrate-producing bacterium A2-183 | AJ270482 |
|  |  | Uncultured bacterium clone Eldhufec122 | AY919997 |
|  |  | butyrate-producing bacterium M72/1 | AY305310 |
|  |  | *Roseburia intestinalis* | AJ312385 |
|  | *Ruminococcus gnavus et rel.* | *Eubacterium contortum* | L34615 |
|  |  | *Ruminococcus gnavus* | L76597 |
|  |  | *Ruminococcus torques* | L76604 |
|  |  | *Clostridium oroticum* | M59109 |
|  |  | *Ruminococcus* sp. CJ60 | AB080891 |
|  |  | uncultured human gut bacterium JW1H4a | AB080862 |
|  |  | uncultured bacterium (human infant) L37A | AF253389 |
|  |  | uncultured bacterium Adhufec117rbh | AY471716 |
|  |  | uncultured bacterium Muc2-3 | AY451997 |
|  | *Ruminococcus hansenii et rel.* | *Ruminococcus productus* | D14144 |
|  |  | *Clostridium coccoides* | M59090 |
|  |  | *Ruminococcus hansenii* | M59114 |
|  |  | *Ruminococcus hydrogenotrophicus* | X95624 |
|  |  | uncultured bacterium KS62 | AY916137 |
|  | *Ruminococcus lactaris et rel.* | bacterium adhufec80.25 | AF153858 |
|  |  | *Ruminococcus lactaris* | L76602 |
|  |  | uncultured bacterium G187 | AY916284 |
|  |  | uncultured bacterium L160 | AY916218 |
|  |  | uncultured bacterium HuRC19 | AY684372 |
|  | *Ruminococcus luti et rel.* | butyrate-producing bacterium T2-132 | AJ270483 |
|  |  | uncultured *Ruminococcus* sp. NO3 | AB064755 |
|  |  | uncultured *Ruminococcus* sp. NB2F4 | AB064753 |
|  |  | uncultured *Ruminococcus* sp. NO2-22 | AB064751 |
|  |  | uncultured bacterium E177 | AY916259 |
|  |  | uncultured bacterium KS90 | AY916138 |
|  |  | uncultured bacterium L068 | AY916217 |
|  |  | uncultured bacterium HuCA5 | AJ408961 |
|  |  | Uncultured bacterium clone Eldhufec106 | AY919981 |
|  |  | Uncultured bacterium UC7-36 | AJ608238 |
|  |  | Uncultured bacterium UC7-7 | AJ608225 |
|  |  | *Ruminococcus luti* | AJ133124 |
|  |  | uncultured bacterium adhufec30.25 | AF153854 |
|  |  | uncultured bacterium Adhufec086abh | AY471687 |
|  |  | uncultured bacterium Adhufec048abh | AY471680 |
|  | *Ruminococcus obeum et rel.* | bacterium adhufec35.25 | AF153853 |
|  |  | *Ruminococcus obeum* | L76601 |
|  |  | uncultured *Ruminococcus* sp. NO67 | AB064763 |
|  |  | uncultured bacterium KZ22 | AY916216 |
|  |  | uncultured bacterium NL49 | AY916245 |
|  |  | uncultured bacterium NQ96 | AY916255 |
|  |  | uncultured bacterium V127 | AY916274 |
|  |  | Uncultured bacterium UC7-35 | AJ608237 |
|  |  | uncultured bacterium Muc1-21 | AY451996 |
|  |  | uncultured bacterium Muc1-11 | AY451995 |
|  |  | uncultured bacterium Muc3-10 | AY452003 |
|  |  | uncultured bacterium Muc3-5 | AY452001 |
|  |  | uncultured bacterium Muc6-16 | AY452019 |
|  |  | uncultured bacterium Muc6-13 | AY452017 |
|  |  | bacterium ucfecDB7 |  |
|  | *Unclutured Ruminococci* | uncultured *Ruminococcus* sp. NS2E3 | AB064750 |
|  |  | uncultured human gut bacterium JW1B11 | AB080869 |
|  |  | uncultured human gut bacterium JW1H7 | AB080868 |
|  |  | uncultured bacterium K379 | AY916198 |
|  |  | uncultured bacterium ME10 | AY916229 |
|  |  | uncultured bacterium HuCB25 | AJ408999 |
|  |  | uncultured bacterium HuCA26 | AJ408977 |
|  |  | uncultured bacterium HuCA2 | AJ408958 |
|  |  | Uncultured bacterium clone Eldhufec132 | AY920007 |
|  |  | Uncultured bacterium clone Eldhufec133 | AY920008 |
|  |  | Uncultured bacterium clone Eldhufec102 | AY919977 |
|  |  | Uncultured bacterium UC7-23 | AJ608235 |
|  |  | uncultured bacterium cadhufec102c10 | AF530364 |
|  |  | uncultured bacterium cadhufec028h7 | AF530301 |
|  |  | uncultured bacterium A20 | AF052417 |
|  |  | uncultured bacterium A14 | AF052415 |
|  |  | uncultured bacterium HuDI20 | AY684379 |
|  |  | uncultured bacterium (human infant) L127 | AF253374 |
|  |  | uncultured bacterium (human infant) P36G | AF253346 |
|  |  | uncultured bacterium (human infant) P36H | AF253344 |
|  |  | uncultured bacterium Adhufec123khh | AY471668 |
|  |  | uncultured bacterium Muc3-9 | AY452002 |
|  |  | uncultured bacterium Muc4-13 | AY452010 |
|  |  | bacterium ucfecDB13 |  |
| *Clostridium* cluster XV | *Eubacterium limosum et rel.* | *Pseudoramibacter alactolyticus* | AB036759 |
|  |  | *Eubacterium limosum* | AF064242 |
|  |  | *Eubacterium barkeri* | M23927 |
|  |  | *Anaerofustis stercorihominis* | AJ518871 |
|  |  | *Eubacterium* sp. CS1 Van | AJ518868 |
| *Clostridium* cluster XVI | *Eubacterium biforme et rel.* | uncultured bacterium D196 | AY916362 |
|  |  | Uncultured bacterium clone Eldhufec204 | AY920079 |
|  |  | Uncultured bacterium clone Eldhufec206 | AY920081 |
|  |  | butyrate-producing bacterium SM7/11 | AY305313 |
|  |  | *Eubacterium biforme* | M59230 |
|  |  | uncultured Gram-positive bacterium NB2C7 | AB064867 |
|  | *Eubacterium cylindroides et rel.* | *Eubacterium cylindroides* | L34616 |
|  |  | *Eubacterium dolichum* | L34682 |
|  |  | *Eubacterium tortuosum* | L34683 |
|  |  | *Clostridium innocuum* | M23732 |
|  | *Solobacterium moorei et rel.* | *Holdemania filiformis* | Y11466 |
|  |  | uncultured bacterium M615 | AY916164 |
|  |  | Uncultured bacterium clone Eldhufec205 | AY920080 |
|  |  | *Solobacterium moorei* | AY044916 |
| *Clostridium* cluster XVII | *Catenibacterium* | *Lactobacillus vitulinus* | M23727 |
|  |  | *Lactobacillus catenaformis* | M23729 |
|  |  | human intestinal firmicute CB12 | AB064934 |
|  |  | Uncultured bacterium clone Eldhufec203 | AY920078 |
|  |  | *Catenibacterium mitsuokai* | AB030226 |
| *Clostridium* cluster XVIII | *Clostridium ramosum et rel.* | *Clostridium cocleatum* | AF028350 |
|  |  | *Clostridium ramosum* | M23731 |
|  |  | *Clostridium spiroforme* | X73441 |
|  |  | Uncultured bacterium clone Eldhufec200 | AY920075 |
|  |  | *Clostridium* sp. 14774 | AJ315981 |
|  | *Coprobacillus catenaformis et rel.* | *Coprobacillus catenaformis* | AB030218 |
|  |  | uncultured bacterium KU74 | AY916140 |
|  |  | uncultured bacterium NI20 | AY916175 |
|  |  | uncultured bacterium LCLC16 | AF499845 |
| Uncultured *Clostridiales* | Uncultured *Clostridiales* I | uncultured human gut bacterium JW2B4 | AB080852 |
|  |  | uncultured bacterium OLDA-F7 | AB099784 |
|  |  | uncultured bacterium OLDB-A9 | AB099783 |
|  |  | uncultured bacterium OLDCA-1 | AB099781 |
|  |  | uncultured bacterium C118 | AY916326 |
|  |  | uncultured bacterium C257 | AY916329 |
|  |  | uncultured bacterium C627 | AY916340 |
|  |  | uncultured bacterium D049 | AY916352 |
|  |  | uncultured bacterium D279 | AY916363 |
|  |  | uncultured bacterium D693 | AY916381 |
|  |  | uncultured bacterium LH65 | AY916208 |
|  |  | uncultured bacterium M220 | AY916150 |
|  |  | uncultured bacterium M233 | AY916151 |
|  |  | uncultured bacterium M412 | AY916156 |
|  |  | uncultured bacterium M621 | AY916165 |
|  |  | uncultured bacterium MF22 | AY916236 |
|  |  | uncultured bacterium MF35 | AY916239 |
|  |  | uncultured bacterium MG86 | AY916291 |
|  |  | uncultured bacterium NH06 | AY916173 |
|  |  | Uncultured bacterium clone Eldhufec312 | AY920187 |
|  |  | Uncultured bacterium clone Eldhufec309 | AY920184 |
|  |  | Uncultured bacterium clone Eldhufec311 | AY920186 |
|  |  | Uncultured bacterium clone Eldhufec308 | AY920183 |
|  |  | Uncultured bacterium clone Eldhufec310 | AY920185 |
|  |  | Uncultured bacterium clone Eldhufec314 | AY920189 |
|  |  | Uncultured bacterium UC7-9 | AJ608227 |
|  |  | Uncultured bacterium UC7-127 | AJ608249 |
|  | Uncultured *Clostridiales* IIa | uncultured human gut bacterium JW2H12 | AB080880 |
|  |  | uncultured bacterium OLDB-C2 | AB099778 |
|  |  | uncultured bacterium C736 | AY916346 |
|  |  | uncultured bacterium LQ86 | AY916269 |
|  |  | uncultured bacterium M501 | AY916160 |
|  |  | Uncultured bacterium clone Eldhufec333 | AY920208 |
|  |  | Uncultured bacterium clone Eldhufec322 | AY920197 |
|  |  | Uncultured bacterium clone Eldhufec332 | AY920207 |
|  | Uncultured *Clostridiales* IIb | uncultured human gut bacterium JW1H11 | AB080881 |
|  |  | uncultured human gut bacterium JW1B2 | AB080879 |
|  |  | uncultured bacterium OLDB-H1 | AB099779 |
|  |  | uncultured bacterium OLDB-F4 | AB099777 |
|  |  | uncultured bacterium C583 | AY916338 |
|  |  | uncultured bacterium C655 | AY916341 |
|  |  | uncultured bacterium D191 | AY916361 |
|  |  | uncultured bacterium K342 | AY916195 |
|  |  | uncultured bacterium M403 | AY916155 |
|  |  | uncultured bacterium MH87 | AY916298 |
|  |  | uncultured bacterium MM92 | AY916304 |
|  |  | uncultured bacterium HuCA6 | AJ408962 |
|  |  | Uncultured bacterium clone Eldhufec328 | AY920203 |
|  |  | Uncultured bacterium clone Eldhufec323 | AY920198 |
|  |  | Uncultured bacterium clone Eldhufec334 | AY920209 |
|  |  | Uncultured bacterium clone Eldhufec330 | AY920205 |
|  |  | Uncultured bacterium clone Eldhufec331 | AY920206 |
|  |  | Uncultured bacterium clone Eldhufec336 | AY920211 |
|  |  | Uncultured bacterium clone Eldhufec327 | AY920202 |
|  |  | Uncultured bacterium clone Eldhufec325 | AY920200 |
|  |  | Uncultured bacterium clone Eldhufec324 | AY920199 |
|  |  | Uncultured bacterium clone Eldhufec326 | AY920201 |
|  |  | uncultured bacterium cadhufec008h7 | AF530296 |
|  |  | uncultured bacterium cadhufec18c08 | AF530351 |
|  |  | uncultured bacterium cadhufec17f05 | AF530343 |
|  |  | uncultured bacterium Adhufec015rbh | AY471695 |
|  |  | uncultured bacterium Adhufec102abh | AY471690 |
|  |  | uncultured bacterium Adhufec123rbh | AY471719 |
| Uncultured *Mollicutes* | Uncultured *Mollicutes* | bacterium adhufec202 | AF132232 |
|  |  | bacterium adhufec279 | AF132233 |
|  |  | uncultured bacterium C027 | AY916325 |
|  |  | uncultured bacterium C133 | AY916328 |
|  |  | uncultured bacterium C611 | AY916339 |
|  |  | uncultured bacterium C754 | AY916348 |
|  |  | uncultured bacterium D051 | AY916353 |
|  |  | uncultured bacterium D423 | AY916369 |
|  |  | uncultured bacterium LW88 | AY916186 |
|  |  | uncultured bacterium MC12 | AY916226 |
|  |  | uncultured bacterium NB12 | AY916191 |
|  |  | Uncultured bacterium clone Eldhufec209 | AY920084 |
|  |  | Uncultured bacterium clone Eldhufec207 | AY920082 |
|  |  | Uncultured bacterium clone Eldhufec208 | AY920083 |
| *Cyanobacteria* | Uncultured *Chroococcales* | uncultured bacterium M019 | AY916143 |
| *Fusobacteria* | *Cetobacterium* | *Cetobacterium somerae* | AJ438155 |
|  | *Fusobacterium* | *Fusobacterium necrophorum* | AF044948 |
|  |  | *Fusobacterium naviforme* | AJ006965 |
|  |  | *Fusobacterium gonidoformans* | M58679 |
|  |  | *Fusobacterium mortiferum* | M58680 |
|  |  | *Fusobacterium varium* | M58686 |
|  |  | *Fusobacterium nucleatum* | X55404 |
|  |  | *Fusobacterium necrogenes* | X55408 |
|  |  | *Fusobacterium russii* | X55409 |
|  |  | *Clostridium rectum* | X77850 |
|  |  | uncultured bacterium HuJJ10 | AY684429 |
|  | *Leptotrichia* | *Leptotrichia bucallis* | L37788 |
| Alpha-Proteobacteria | *Methylobacterium* | uncultured bacterium ABLCf14 | AF499910 |
|  | *Novosphingobium* | uncultured bacterium ABLCf85 | AF499911 |
|  | *Oceanospirillum* | uncultured bacterium D623 | AY916377 |
|  |  | uncultured bacterium D784 | AY916388 |
|  |  | uncultured bacterium MK72 | AY916302 |
|  |  | uncultured bacterium V326 | AY916278 |
| Beta-Proteobacteria | *Alcaligenes faecalis et rel.* | *Achromobacter denitrificans* | AF232712 |
|  |  | uncultured bacterium ABLC15 | AF499888 |
|  |  | *Alcaligenes faecalis* | DQ110882 |
|  |  | *Kerstersia gyiorum* | AY131213 |
|  | *Aquabacterium* | uncultured bacterium ABLC71 | AF499885 |
|  | *Burkholderia* | uncultured bacterium LCLC40 | AF499842 |
|  | *Neisseria* | uncultured bacterium HuJJ55 | AY684428 |
|  | *Oxalobacter formigenes et rel.* | *Oxalobacter formigenes* | U49749 |
|  |  | uncultured bacterium ABLC55 | AF499887 |
|  | *Sutterella wadsworthia et rel.* | *Sutterella wadsworthia* | L37785 |
|  |  | uncultured bacterium D093 | AY916355 |
|  |  | uncultured bacterium M105 | AY916147 |
|  |  | uncultured bacterium HuCA4 | AJ408960 |
|  |  | uncultured bacterium HuCC33 | AJ315485 |
|  |  | Uncultured bacterium clone Eldhufec064 | AY919939 |
|  |  | Uncultured bacterium clone Eldhufec063 | AY919938 |
|  |  | uncultured bacterium ABLC72 | AF499889 |
|  |  | uncultured bacterium HuDI12 | AY684426 |
| Gamma-Proteobacteria |  | *Aeromonas veronii* | AF099024 |
|  | *Aeromonas* | *Aeromonas enteropelogenes* | S42871 |
|  | *Anaerobiospirillum* | *Anaerobiospirillum thomasii* | AJ420985 |
|  |  | *Anaerobiospirillum succiniciproducens* | U96412 |
|  | *Enterobacter aerogenes et rel.* | *Enterobacter aerogenes* | AB004750 |
|  |  | *Citrobacter freundii* | AF025365 |
|  |  | *Citrobacter koseri* | AF025366 |
|  |  | *Citrobacter braakii* | AF025368 |
|  |  | *Citrobacter werkmanii* | AF025373 |
|  |  | *Tatumella ptyseos* | AJ233437 |
|  |  | *Raoultella terrigena* | Y17658 |
|  |  | *Klebsiella oxytoca* | Y17660 |
|  |  | *Raoultella planticola* | Y17663 |
|  |  | *Enterobacter cancerogenus* | Z96078 |
|  |  | *uncultured bacterium OLDA-E9* | AB099791 |
|  |  | *Citrobacter gillenii* | AF025367 |
|  |  | *Citrobacter murliniae* | AF025369 |
|  |  | *Averyella dalhousiensis* | DQ481464 |
|  | *Escherichia coli et rel.* | *Escherichia coli* | A14565 |
|  |  | *Edwardsiella tarda* | AF015259 |
|  |  | *Citrobacter sedlakii* | AF025364 |
|  |  | *Citrobacter farmeri* | AF025371 |
|  |  | *Salmonella enterica* | U90318 |
|  |  | *Shigella flexneri* | X80679 |
|  |  | *Shigella dysenteriae* | X80680 |
|  |  | *Uncultured bacterium clone Eldhufec069* | AY919944 |
|  |  | *Cedecea davisae* | AF493976 |
|  |  | *Escherichia fergusonii* | AF530475 |
|  |  | *Trabulsiella guamensis* | AY373830 |
|  |  | *Citrobacter amalonaticus* | AF025370 |
|  |  | *uncultured bacterium Muc4-17* | AY452011 |
|  | *Haemophilus* | *Haemophilus haemolyticus* | M75045 |
|  |  | *Haemophilus parainfluenzae* | M75081 |
|  | *Klebsiella pneumoniae et rel.* | *Pantoea agglomerans* | AB004691 |
|  |  | *Serratia liquefaciens* | AB004752 |
|  |  | *Klebsiella pneumoniae* | AB004753 |
|  |  | *Enterobacter cloacae* | AF157695 |
|  |  | *Yokenella regensburgei* | AY269192 |
|  |  | *Enterobacter asburiae* | AB004744 |
|  | *Leminorella* | *Leminorella grimontii* | AJ233421 |
|  | *Moraxellaceae* | *Moraxella catarrhalis* | A27627 |
|  |  | *Acinetobacter calcoaceticus* | AF159045 |
|  |  | *Acinetobacter johnsonii* | AF188300 |
|  |  | *Acinetobacter haemolyticus* | Z93437 |
|  |  | *uncultured bacterium HuJJ26* | AY684425 |
|  |  | *uncultured bacterium HuJJ19* | AY684423 |
|  | *Proteus et rel.* | *Providencia stuartii* | AF008581 |
|  |  | *Proteus mirabilis* | AF008582 |
|  |  | *Proteus vulgaris* | AJ233425 |
|  |  | *Morganella morganii* | AJ301681 |
|  |  | *Providencia alcalifaciens* | AJ301684 |
|  |  | *Providencia rettgeri* | AM040492 |
|  |  | *Providencia rustigianii* | AM040489 |
|  |  | *Moellerella wisconsensis* | AM040754 |
|  |  | *Proteus penneri* | AJ634474 |
|  | *Pseudomonas* | *Pseudomonas aeruginosa* | AB037545 |
|  |  | *Pseudomonas stutzeri* | AF038653 |
|  | *Pseuodomonas* | *Pseudomonas monteilii* | AF064458 |
|  |  | *Pseudomonas fluorescens* | AJ278813 |
|  |  | *Pseudomonas putida* | D84020 |
|  | *Serratia* | *Serratia marcescens* | M59160 |
|  | *Vibrio* | *Vibrio parahaemolyticus* | M59161 |
|  |  | *Grimontia hollisae* | S83393 |
|  |  | *Vibrio fluvialis* | X74703 |
|  |  | *Vibrio furnissii* | X74704 |
|  | *Xanthomonadaceae* | uncultured bacterium ABLCf21 | AF499898 |
|  |  | uncultured bacterium ABLC16 | AF499891 |
|  | *Yersinia et rel.* | *Yersinia pseudotuberculosis* | AF282307 |
|  |  | *Yersinia enterocolitica* | AF282308 |
|  |  | *Hafnia alvei* | M59155 |
|  |  | *Yersinia frederiksenii* | X75273 |
|  |  | *Yersinia rohdei* | X75276 |
|  |  | *Yersinia kristensenii* | X75278 |
|  |  | *Yersinia bercovieri* | X75281 |
| Delta-  Proteobacteria | *Bilophila* | *Bilophila wadsworthia* | L35148 |
|  | *Desulfovibrio et rel.* | *Desulfovibrio desulfuricans* | AF098671 |
|  |  | *Desulfvibrio piger* | AF192152 |
|  |  | uncultured bacterium D168 | AY916359 |
|  |  | uncultured bacterium LE30 | AY916206 |
|  |  | Uncultured bacterium clone Eldhufec073 | AY919948 |
|  |  | *Desulfovibrio fairfieldensis* | U42221 |
|  |  | bacterium ucfecDB10 |  |
|  |  | bacterium ucfecDB12 |  |
| Epsilon-Proteobacteria | *Arcobacter* | *Arcobacter cryaerophilus* | L14624 |
|  |  | *Arcobacter butzleri* | U34386 |
|  | *Campylobacter* | *Campylobacter hominis* | AF062490 |
|  |  | *Campylobacter fetus* | AJ306568 |
|  |  | *Campylobacter jejuni* | AL139074 |
|  |  | *Campylobacter coli* | L04312 |
|  |  | *Campylobacter lari* | L04316 |
|  |  | *Campylobacter rectus* | L04317 |
|  |  | *Campylobacter gracilis* | L04320 |
|  |  | *Bacteroides ureolyticus* | L04321 |
|  |  | *Campylobacter concisus* | L04322 |
|  |  | *Campylobacter upsaliensis* | L14628 |
|  | *Helicobacter* | *Helicobacter pylori* | AE000511 |
|  |  | *Flexispira rappini* | AF034135 |
|  |  | *Helicobacter canadensis* | AF262037 |
|  |  | *Helicobacter cinaedi* | AF396082 |
|  |  | *Helicobacter pullorum* | L36141 |
|  |  | *Helicobacter winghamensis* | AF246984 |
| *Lentisphaerae* | *Victivallis* | *Victivallis vadensis* | AY049713 |
| *Spirochaetes* | *Brachyspira* | *Brachyspira aalborgi* | AF395882 |
|  |  | *Brachyspira pilosicoli* | AY155458 |
| *Verruco-microbia* | *Akkermansia* | Uncultured bacterium clone Eldhufec002 | AY919877 |
|  |  | *Akkermansia muciniphila* | AY271254 |
|  |  | uncultured bacterium HuRC51 | AY684431 |
