# Supplementary material for: Development and application of the human intestinal tract chip, a phylogenetic microarray: analysis of universally conserved phylotypes in the abundant microbiota of young and elderly adults
Source: Environ Microbiol. 2009 Jul;11(7):1736–51. doi: 10.1111/j.1462-2920.2009.01900.x (PMC2784037; doi:10.1111/j.1462-2920.2009.01900.x)
Supplement: Supplementary file 6 [file emi0011-1736-SD6.doc]

**Table S2** Pairs of human gastrointestinal species and yet uncultured phylotypes that can not be distinguished based on their variable region V1 and V6 SSU rRNA gene sequence.

| **Species** | **GenBank accession number** | **Percentage of overall SSU rRNA gene similarity** |
| --- | --- | --- |
| *Bifidobacterium* sp. PL1 | AF306789 | 98.38 |
| uncultured *Bifidobacterium* sp.15D | AF275886 |
| *Bifidobacterium breve* | AB006658 | 97.69 |
| *Bifidobacterium infantis* | D86184 |
| *Bacillus flexus* | AB021185 | 98.79 |
| *Bacillus megaterium* | D16273 |
| *Enterococcus casseliflavus* | AF039899 | 99.86 |
| *Enterococcus gallinarum* | AF039898 |
| *Eubacterium budayi* | AB018183 | 99.32 |
| *Clostridium barati* | X68174 |
| *Clostridium paraperfringens* | M59102 | 98.94 |
| *Clostridium paraputrificum* | AB032556 |
| Uncultured bacterium HUCA2 | AJ408958 | 98.96 |
| Uncultured bacterium HUCA26 | AJ408977 |
| *Citrobacter braakii* | AF025368 | 99.74 |
| *Citrobacter freundii* | AB006658 |
| *Raoultella planticola* | Y17663 | 99.09 |
| *Enterobacter aerogenes* | AB004750 |
| *Klebsiella pneumoniae* | AB004753 | 99.37 |

**Table S3.** Phylogenetic identification of core probes

| **Probe specificity** | **Absolute number and relative frequency (%) of the core HITChip probes** | |
| --- | --- | --- |
|  | **Younger** | **Elderly** |
| *Actinobacteria* | 22 (3.1) | 12 (3.4) |
| *Bacteroidetes* | 122 (17.2) | 40 (11.4) |
| *Bacilli* | 25 (3.5) | 11 (3.1) |
| *Clostridium* cluster I | 4 (0.6) | 0 |
| *Clostridium* cluster III | 3 (0.4) | 1 (0.9) |
| *Clostridium* cluster IV | 142 (20) | 88 (25.1) |
| *Clostridium* cluster IX | 1 (0.1) | 0 |
| *Clostridium* cluster XI | 19 (2.7) | 5 (1.4) |
| *Clostridium* cluster XIVa | 330 (46.5) | 170 (48.6) |
| *Clostridium* cluster XIV | 1 (0.1) | 2 (0.6) |
| *Clostridium* cluster XVI | 5 (0.7) | 4 (1.1) |
| Uncultured *Clostridiales* | 1 (0.1) | 5 (1.4) |
| Beta-*Proteobacteria* | 8 (1.1) | 3 (0.9) |
| Gamma-*Proteobacteria* | 4 (0.6) | 0 |
| Delta-*Proteobacteria* | 1 (0.1) | 0 |
| Universal | 22 (3.1) | 9 (2.6) |
